# Bioengineered human pseudoislets form efficiently from donated tissue, compare favourably with native islets in vitro and restore normoglycaemia in mice

**DOI:** 10.1007/s00125-018-4672-5

**Published:** 2018-07-03

**Authors:** Yang Yu, Anissa Gamble, Rena Pawlick, Andrew R. Pepper, Bassem Salama, Derek Toms, Golsa Razian, Cara Ellis, Antonio Bruni, Boris Gala-Lopez, Jia (Lulu) Lu, Heather Vovko, Cecilia Chiu, Shaaban Abdo, Tatsuya Kin, Greg Korbutt, A. M. James Shapiro, Mark Ungrin

**Affiliations:** 1 Biomedical Engineering Graduate Program, University of Calgary, Calgary, AB Canada; 2 Alberta Diabetes Institute, University of Alberta, Edmonton, AB Canada; 3 Canadian National Transplant Research Program, Edmonton, AB Canada; 4 Department of Comparative Biology and Experimental Medicine, Faculty of Veterinary Medicine, University of Calgary, Heritage Medical Research Building Room 412, 3330 Hospital Drive NW, Calgary, AB T2N 4N1 Canada; 5 Clinical Islet Transplant Program, University of Alberta, Edmonton, AB Canada; 6 Alberta Children’s Hospital Research Institute, University of Calgary, Calgary, AB Canada

**Keywords:** Hypoxia, Islet, Mouse, Pseudoislet, Transplant

## Abstract

**Aims/hypothesis:**

Islet transplantation is a treatment option that can help individuals with type 1 diabetes become insulin independent, but inefficient oxygen and nutrient delivery can hamper islet survival and engraftment due to the size of the islets and loss of the native microvasculature. We hypothesised that size-controlled pseudoislets engineered via centrifugal-forced-aggregation (CFA-PI) in a platform we previously developed would compare favourably with native islets, even after taking into account cell loss during the process.

**Methods:**

Human islets were dissociated and reaggregated into uniform, size-controlled CFA-PI in our microwell system. Their performance was assessed in vitro and in vivo over a range of sizes, and compared with that of unmodified native islets, as well as islet cell clusters formed by a conventional spontaneous aggregation approach (in which dissociated islet cells are cultured on ultra-low-attachment plates). In vitro studies included assays for membrane integrity, apoptosis, glucose-stimulated insulin secretion assay and total DNA content. In vivo efficacy was determined by transplantation under the kidney capsule of streptozotocin-treated *Rag1*^−/−^ mice, with non-fasting blood glucose monitoring three times per week and IPGTT at day 60 for glucose response. A recovery nephrectomy, removing the graft, was conducted to confirm efficacy after completing the IPGTT. Architecture and composition were analysed by histological assessment via insulin, glucagon, pancreatic polypeptide, somatostatin, CD31 and von Willebrand factor staining.

**Results:**

CFA-PI exhibit markedly increased uniformity over native islets, as well as substantially improved glucose-stimulated insulin secretion (8.8-fold to 11.1-fold, even after taking cell loss into account) and hypoxia tolerance. In vivo, CFA-PI function similarly to (and potentially better than) native islets in reversing hyperglycaemia (55.6% for CFA-PI vs 20.0% for native islets at 500 islet equivalents [IEQ], and 77.8% for CFA-PI vs 55.6% for native islets at 1000 IEQ), and significantly better than spontaneously aggregated control cells (55.6% for CFA-PI vs 0% for spontaneous aggregation at 500 IEQ, and 77.8% CFA-PI vs 33.4% for spontaneous aggregation at 1000 IEQ; *p* < 0.05). Glucose clearance in the CFA-PI groups was improved over that in the native islet groups (CFA-PI 18.1 mmol/l vs native islets 29.7 mmol/l at 60 min; *p* < 0.05) to the point where they were comparable with the non-transplanted naive normoglycaemic control mice at a low IEQ of 500 IEQ (17.2 mmol/l at 60 min).

**Conclusions/interpretation:**

The ability to efficiently reformat dissociated islet cells into engineered pseudoislets with improved properties has high potential for both research and therapeutic applications.

**Electronic supplementary material:**

The online version of this article (10.1007/s00125-018-4672-5) contains peer-reviewed but unedited supplementary material, which is available to authorised users.

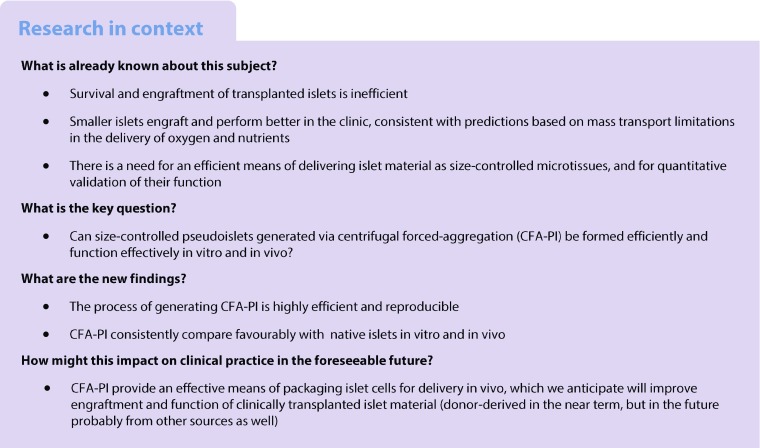



## Introduction

Type 1 diabetes is a major health problem affecting tens of millions of individuals, and its prevalence has been increasing globally [1, 2]. Conventional insulin-replacement therapies are only palliative and fail to correct excursions in glycaemic control, with consequent secondary diabetic complications of blindness, kidney failure, limb amputation, heart attack, stroke and up to a 15 year reduction in life expectancy [3, 4].

Islet transplantation represents a promising and effective treatment approach in selected individuals with risk of hypoglycaemia [5–7], but this treatment currently fails to provide a cure. Substantial islet loss in the immediate post-transplant period results from ischaemia and an instant blood-mediated inflammatory response, coupled with poor vascularisation after implantation [8]. The islet microvasculature is disconnected during isolation and degenerates during culture, and the post-transplant process of recruiting host endothelial cells is slow, even more so in the human system than the mouse [8]. Prior to revascularisation, delivery of oxygen and nutrients occurs only via diffusion, which is insufficient to support cells in the core of the islet [9, 10]. Even after a prolonged period, the native vasculature is not fully restored [11–13], which is likely to contribute to long-term islet loss due to chronic stress [14], and reduced oxygen levels negatively affect function [15].

A widely researched approach to achieve immune tolerance in islet transplantation is to conceal the transplanted material from the host immune system using encapsulation [16], and strategies targeting improved islet survival and engraftment must be compatible with this concept. This approach provides an additional diffusive barrier to the flow of nutrients and oxygen, reducing delivery to the islet and consequently the slope of the concentration gradient driving delivery to cells in the interior [10]. Furthermore, in fulfilling the objective of blocking access by cells of the host immune system to the transplanted tissue, the capsule also prevents access by the host endothelium, permanently forestalling reconstitution of the microvasculature and full integration with the host blood system. In this case, engrafted islets will be completely dependent on the diffusive delivery of oxygen and nutrients for the duration of the graft. Quantitative modelling of oxygen delivery shows significant benefits to smaller islets (whether encapsulated or not) [10, 17], and, consistent with this concept, smaller human islets are reported to perform better than larger ones both in a clinical setting [18] and in culture [19]. There is thus potential for engineering size-controlled islet cell clusters (‘pseudoislets’) to overcome some of these challenges.

Many attempts have been made to dissociate native islets and reaggregate them [20–26]. The most common method has employed spontaneous aggregation, where the dissociated islet cell suspension is cultured in ultra-low-binding plates [26–28]. This method results in pseudoislets that are heterogeneous in size, and yields are low. Alternatively, a hanging-drop approach has been used to form pseudoislets from rat [29] and human islet [30] sources. Although this approach yields pseudoislets of uniform size, the method is labour intensive and difficult to scale. More recently, there have been several reports of the formation of pseudoislets using a microwell technique [31–33]. These approaches have, however, relied on a variety of customised devices, which restricts widespread reproduction of the work and may impose limits on scalability. Moreover, the clinical relevance and quantitative in vivo and in vitro performance have not yet been fully investigated.

We have previously established a scalable microwell platform for the generation of large numbers of uniform cellular aggregates [34], now widely available under the AggreWell name. In the present study, we apply a scalable centrifugal-forced-aggregation approach to generate large numbers of uniform, size-controlled pseudoislets, and characterise them in vitro and in vivo.

## Methods

For detailed Methods, please refer to electronic supplementary material (ESM) Methods.

### Human islet isolation

Human islet preparations were provided by the Alberta Diabetes Institute IsletCore and the Clinical Islet Transplant Program at the University of Alberta in Edmonton, under approvals for the use of human tissue from the respective Health Research Ethics Boards at both the University of Alberta and the University of Calgary.

### Human islet dissociation and reaggregation

Islets were dissociated in TrypLE Select (Thermo Fisher Scientific, Waltham, MA, USA) at 37°C in a shaking water bath for 8–10 min, followed by trituration to break up remaining clumps, and formed into pseudoislets engineered via centrifugal-forced-aggregation (CFA-PI) in 24-well or 6-well AggreWell 400 plates (STEMCELL Technologies, Vancouver, BC, Canada).

### Static glucose-stimulated insulin secretion

A static glucose-stimulated insulin secretion (GSIS) assay was performed for precultured native islets and dissociated single cells (ESM Fig. 1), and after 3–5 days of culture for all groups at 200 islet equivalents (IEQ) per group. The islets were washed of residual glucose three times in glucose-free medium, and incubated in RPMI-1640 containing low (2.8 mmol/l) glucose for 1 h, followed by high (16.7 mmol/l) glucose for an additional hour at 37°C and 5% CO_2_. The supernatant fraction was harvested after each glucose incubation and insulin levels were measured by ELISA (Cat. no. 10-1113-01; Mercodia, Uppsala, Sweden).

### Hypoxic culture and viability analysis

CFA-PI and islets were cultured at 37°C in 5% CO_2_ for 7 days in both ambient and hypoxic (5% O_2_ incubator) conditions. Islet and CFA-PI viability was then assessed using the inclusion and exclusion dyes fluorescein diacetate A and propidium iodide (PI) [35].

### Apoptosis TUNEL staining

 Apoptosis of islets and pseudoislets was assessed by TUNEL assay (Promega, Madison, WI, USA).

### Quantitative real-time reverse transcription PCR

RNA was isolated using a Total RNA Purification Kit (Norgen Biotek, Thorold, ON, Canada), quantified on a NanoDrop 2000 spectrophotometer (Thermo Fisher Scientific), and reverse transcribed using the iScript Reverse Transcription Supermix (Bio-Rad, Hercules, CA, USA). Quantitative real-time reverse transcription PCR (qRT-PCR) was carried out (see ESM Table 1 for primer sequences) on an Applied Biosystems Cycler (Thermo Fisher Scientific), and analysed with the ∆C_t_ method [36] with normalisation against two stable internal reference genes (*POLR2A* and *EIF2B1*) [37]. Genes analysed were grouped into four categories—oxidative defence: *SOD1*, *SOD2*, *CAT*; secretory function: *INS*, *PDX1*, *GLP1R*, *PCSK1*, *PCSK2*; cell communication: *GJA1*, *CDH1*, *LAMB1*, *ITGB1*, *ITGB7*; and apoptosis: *NFKB1*, *NOS2*, *NOS3*, *MAPK8*, *MAPK10*, *APAF1.*

### Islet transplantation

Female and male adult (8–10 weeks of age, 20–30 g) immunodeficient mice (B6.129S7-*Rag1*^tm1Mom/J^) were obtained from Jackson Laboratories (Bar Harbor, ME, USA). The care of the animals was in accordance with the guidelines approved by the Canadian Council on Animal Care. All in vivo experiments were carried out at the Alberta Diabetes Institute under approvals from the Research Ethics Board of the University of Alberta. Animals were housed in cages with no more than five animals per cage in a temperature-controlled environment, on a light/dark cycle with ad libitum access to food and water. Animals were streptozotocin-induced and considered diabetic after two consecutive non-fasting blood glucose measurements ≥18 mmol/l. Animals that did not become hyperglycaemic after induction were excluded. Animals were block randomised to ensure a balance of all groups per isolation and all groups within cages. Native islets, CFA-PI (divided into pseudoislet [P] 500, P750 and P1000 groups, formed from 1000 cells, 750 cells and 500 cells, respectively) and spontaneous aggregates were transplanted under the capsule of the left kidney in diabetic mice. Islet graft function was assessed using non-fasting blood glucose measurements, three times per week for 60 days; at this point the graft was retrieved from normoglycaemic mice (<11.1 mmol/l) via a recovery nephrectomy of the left kidney, and reversion to hyperglycaemia was confirmed, ≥18 mmol/l.

### IPGTTs

In vivo glucose tolerance and islet function in mice regardless of euglycaemia were assessed by IPGTT 60 days post-transplant. The mice were fasted overnight, and 25% dextrose (Hospira, Lake Forest, IL, USA) was administered intraperitoneally at 3 g/kg body weight. Animal group identifications were blinded and blood glucose measurements were monitored at baseline (*t* = 0), 15, 30, 60, 90 and 120 min.

### Immunohistochemistry

Islet and CFA-PI transplant grafts were removed from mice, fixed in 10% formalin, embedded in paraffin and sectioned. Following de-paraffinisation and antigen heat retrieval, sections were blocked with 20% goat serum (Sigma-Aldrich, St. Louis, MO, USA) in DPBS for 1.5 h at room temperature. For vessel staining, an additional enzymatic antigen retrieval step was performed prior to blocking (proteinase K at 20 μg/ml for 20 min at 37°C). Sections were incubated with primary antibodies overnight at 4°C and secondary antibodies for 1 h at room temperature, and counterstained with DAPI (Invitrogen, Carlsbad, CA, USA).

### Statistical analysis

Group assignment of in vivo study was blinded to the surgeons. Groups were blinded during the collection of the blood glucose measurements. In vitro study was also blinded, except for group labelling of supernatant collection in GSIS assays. No sample was excluded from in vitro data due to low performance. Two mice were excluded from the in vivo data, one died at day 3 post-transplant, and one did not become hyperglycaemic after recovery nephrectomy. Data are expressed as mean ± SEM unless otherwise specified. Statistical analyses were performed using GraphPad Prism (version 7.0, https://www.graphpad.com/scientific-software/prism). Efficacy ratio analysis was conducted through one-way ANOVA. Stimulation index and TUNEL analyses were conducted through Kruskal–Wallis test. qRT-PCR analysis was conducted through Mann–Whitney *U* test. Comparison of euglycaemia curves was by Gehan–Breslow–Wilcoxon test. IPGTT analysis was conducted through unpaired two-tailed *t* test between groups at each time point. AUC analysis of IPGTT was conducted through one-way ANOVA. Total area analyses of endocrine markers and blood vessels were conducted through unpaired two-tailed *t* test. Imaging was carried out on a Zeiss Colibri inverted fluorescence microscope (ZEISS, Oberkochen, Germany) unless otherwise specified, and analysis was via ImageJ software (version 1.51r, https://imagej.nih.gov/ij). Spatial statistical analysis of vascular element distribution was performed using the Spatial statistics 2D/3D plugin (version 3.9, http://imagejdocu.tudor.lu/doku.php?id=plugin:analysis:spatial_statistics_2d_3d:start#download), implementing the previously published F-function [38, 39], with independent evaluation points set to 10,000, hardcore distance set to 0 and pattern samples set to 10.

## Results

### CFA-PI form effectively and show substantially increased geometric consistency compared with native islets

**Fig. 1:**
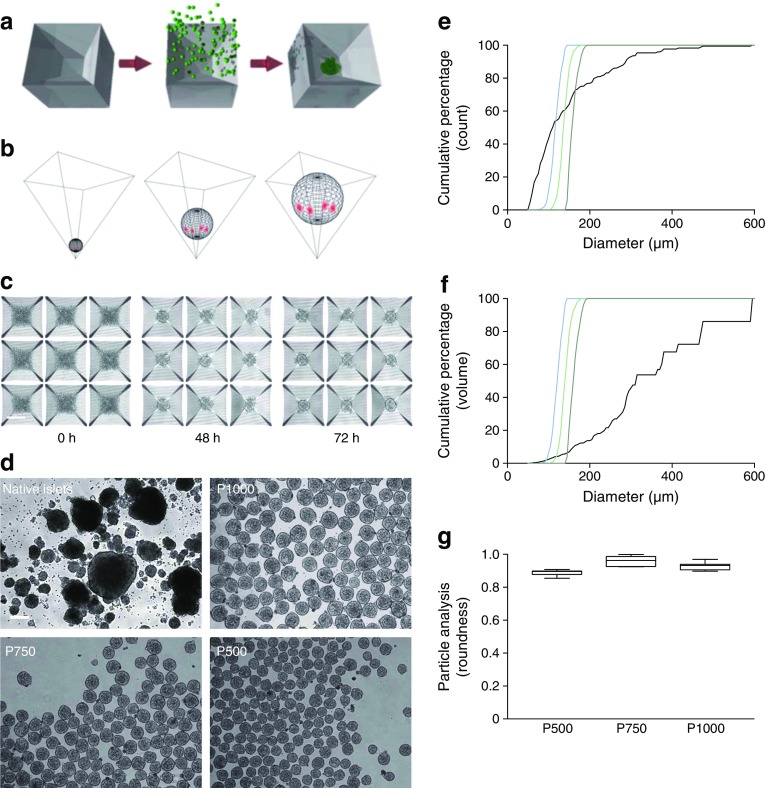
(**a**) CFA-PI are formed via centrifugation of a suspension of single cells into square-pyramidal microwells, where they adhere to one another and collapse into a spherical configuration. (**b**) As the pyramidal shape is precise to sub-micrometre resolution, the geometric relationship between the aggregate, the microwell and the underlying medium space (red highlights indicate contact points) is independent of aggregate size. CFA-PI form over 48–72 h (**c**) in 400 μm microwells (scale bars, 200 μm) and exhibit controlled sizes (P1000, P750 and P500) and enhanced consistency over native islets (**d**). (**e**) Quantifying these images, the cumulative size distribution of CFA-PIs emphasises the dramatic improvement in consistency compared with native islets (black line). (**f**) We also modelled mass distribution vs size (cumulative proportion of total volume contained in structures of a given size or smaller). Although this should be considered only as an approximation as true volumetric data were not collected (volume was modelled as a sphere of the measured radius, which is true for CFA-PI but less so for islets), these differences, as well as the pronounced degree to which islet cells are more likely to be found in large islets, are further emphasised. (**e**, **f**) Dark green line, P1000; light green line, P750; blue line, P500; black line, native islets. (**g**) Sphericity of CFA-PI was confirmed by quantifying roundness (defined as 4 × [Area] / (π × [Major axis]^2^) of randomly selected CFA-PI imaged before and after 90° rotation (*n* = 10) using a micromanipulator (roundness = 1.0 indicates perfectly round aggregates)

CFA-PI form and coalesce into spherical structures (Fig. 1a–c), as expected from well-understood free-energy-minimisation models [40, 41]. Varying the number of input cells provided precise control over CFA-PI size, and markedly enhanced symmetry and size consistency compared with native islets (Fig. 1d–f). Comparison of diameter in all three axes via micromanipulation of individual CFA-PI confirmed that this symmetry extended to all three dimensions (Fig. 1g). In the process, we were also able to confirm the accessibility of the individual cells prior to CFA-PI assembly for genetic modification (ESM Fig. 2), which may prove useful in future applications.

### CFA-PI compare favourably with native islets in vitro

The selection of a suitable readout is vital for bioprocess assessment. In the case of islet transplantation for the treatment of insulin-dependent diabetes, the primary constraint is a shortage of transplantable pancreatic islet material, while the desired product is the capacity to secrete insulin upon exposure to glucose. Accordingly, we developed a quantitative attribute, termed the efficacy ratio, based on cell numbers and static GSIS results, which represents the amount of glucose-regulated insulin secretion obtained from a known quantity of starting material:

$$ \mathrm{Efficacy}\ \mathrm{ratio}=\left(\frac{\mathrm{Cells}\ \mathrm{per}\ \mathrm{pseudoislet}}{\mathrm{Input}\ \mathrm{cells}\ \mathrm{per}\ \mathrm{pseudoislet}}\right)\times \left(\frac{\mathrm{Insulin}\ \mathrm{production}}{\mathrm{Number}\ \mathrm{of}\ \mathrm{cells}\ \mathrm{assayed}}\right) $$

This quantity represents the amount of insulin-generating capacity that results per primary pancreatic islet cell used in the process.

**Fig. 2:**
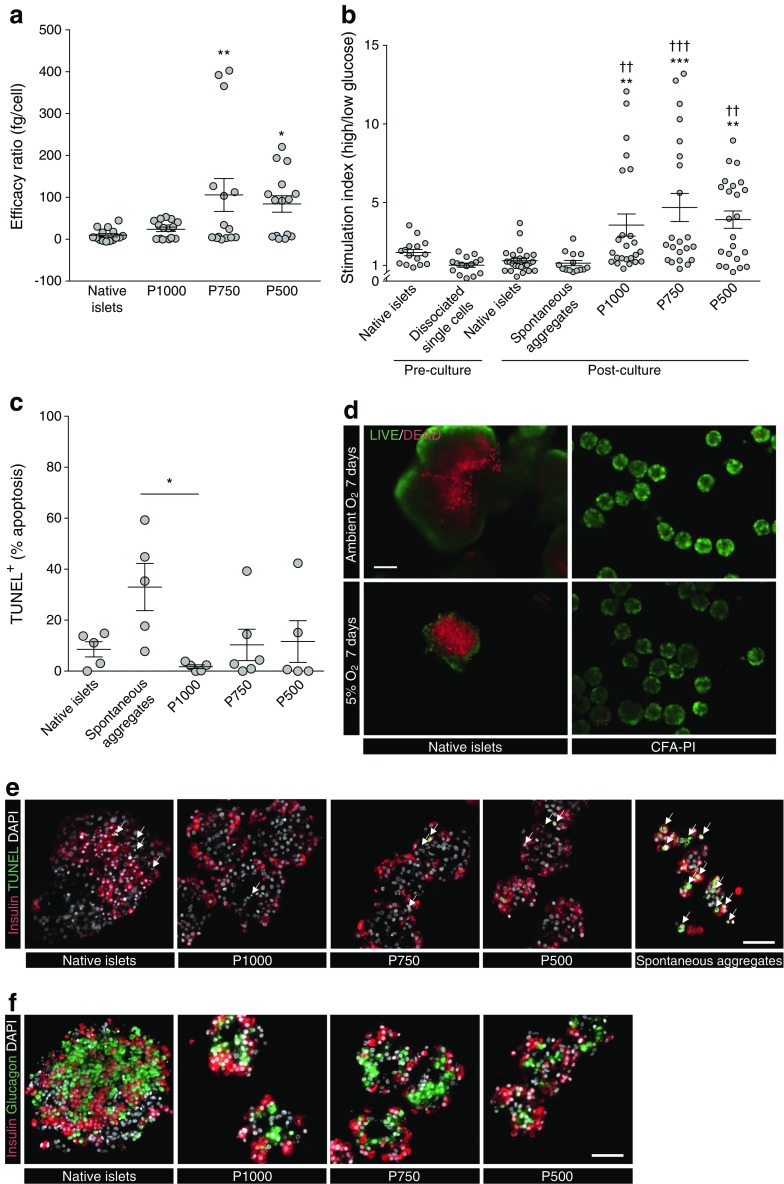
(**a**) Overall CFA-PI showed enhanced GSIS per input cell, and the efficacy ratio for P750 and P500 pseudoislets was significantly improved compared with native islets: 105.70 ± 39.24 fg/cell and 84.19 ± 19.79 fg/cell vs 9.53 ± 3.87 fg/cell; *p < 0.05, **p < 0.01; *n* = 15 per group, one-way ANOVA with Dunnett’s correction for multiple comparisons. (**b**) Stimulation index (insulin secretion under high glucose divided by secretion under low glucose) was assessed for islets upon receipt and immediately following dissociation to single cells, and after 3–5 days of culture as islets, spontaneous aggregates and CFA-PI. CFA-PI showed significant improvements over both native islets (**p < 0.01, ***p < 0.001) and spontaneous aggregates (††*p* < 0.01, †††*p* < 0.001); data presented as mean ± SEM, *n* ≥ 12 per group, Kruskal–Wallis test with Dunn’s correction for multiple comparisons. (**c**) TUNEL staining after 3–5 days in culture showed significantly increased apoptosis in the spontaneous aggregates compared with the P1000 CFA-PI (33.0 ± 9.2% vs 1.8 ± 0.7%; *p < 0.05; *n* = 5, Kruskal–Wallis test), other comparisons were not statistically significant. (**d**) Representative images show differences in cell death in native islets and CFA-PI cultured for 7 days under ambient and hypoxic (5% O_2_) atmospheric conditions and stained for live (green) and dead (red) cells (scale bar, 100 μm). (**e**) Immunostaining of cultured native islets, CFA-PI (P1000, P750 and P500) and spontaneous aggregates prior to transplant for insulin (red), TUNEL^+^ (green/arrows) and nuclei (DAPI, grey) shows the increased apoptosis in spontaneous aggregates. (**f**) Immunolocalisation of insulin (red) and glucagon (green) shows potential structural differences in the location of alpha and beta cells between native islets (intermingled) and CFA-PI (beta cells at periphery) (scale bars, 50 μm)

When comparing the efficacy ratio of CFA-PI with that of native islets, we observed a trend of increase in CFA-PI groups in which the improvements with P500 and P750 were statistically significant, exhibiting an 8.8- and 11.1-fold increase over native islets (Fig. 2a). Comparing the stimulation index of CFA-PI with that of native islets and spontaneous aggregates, we found a significant increase for P1000, P750 and P500 compared with native islets and spontaneous aggregates (Fig. 2b). CFA-PI retained their stimulation index advantage over native islets for at least 15 days under standard culture conditions (ESM Fig. 3). Additional data on insulin release in response to glucose stimulation are presented in ESM Figs 4–7 and ESM Tables 2–7.

The effects of hypoxic culture on CFA-PI and native islets were assessed after a 7-day culture period. Viability estimation using exclusion and inclusion fluorescent dyes (fluorescein diacetate/propidium iodide) revealed substantially higher cell death in native islets even under ambient atmospheric conditions, in sharp contrast to CFA-PI, and this effect was further exacerbated when the cells were cultured in a 5% O_2_ atmosphere (Fig. 2d).

There was no significant difference in the percentage of apoptotic cells (TUNEL assay) between CFA-PI and native islets cultured under standard conditions; however, spontaneous aggregates showed a statistically significant increase in apoptosis compared with the CFA-PI P1000 group (Fig. 2c,e). Immunolocalisation of insulin- and glucagon-positive cells within native islets was heterogeneous, whereas CFA-PI exhibited a peripheral localisation of insulin-positive cells around a core composed primarily of glucagon-positive cells (Fig. 2f).

### CFA-PI show similar gene expression profiles to native islets

**Fig. 3:**
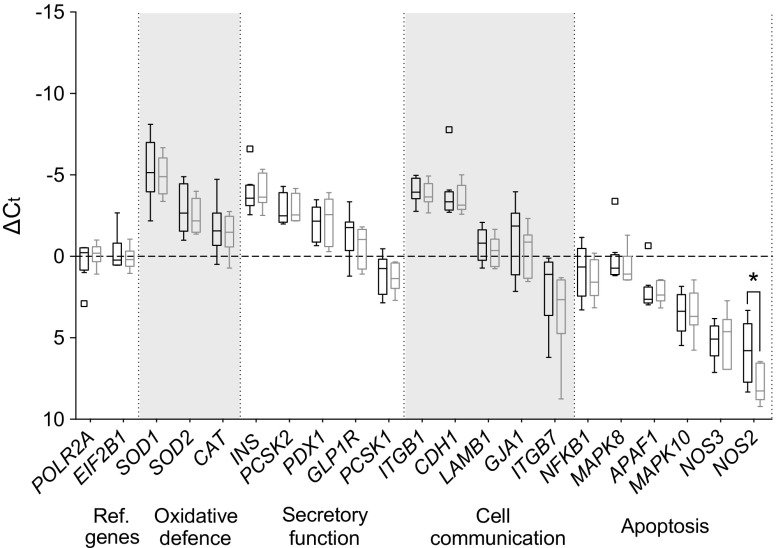
qRT-PCR was performed to identify differences between the gene expression profiles (in four categories, with alternate grey and white shading) of native islets (*n* = 8) and P750 CFA-PI (*n* = 7) 48 h after aggregation. Results were normalised to the reference (Ref.) genes *POLR2A* and *EIF2B1* and are presented as ΔC_t_ values to allow comparison between native islets (black) and CFA-PI (grey) on a gene-by-gene basis. Data are plotted in Tukey box-and-whisker format, where the boxes indicate the interquartile range, with an internal horizontal line indicating the median. Outliers (defined as points outside 3/2 the interquartile range) are plotted individually, and the ‘whisker’ lines indicate data range from minimum to maximum excluding outliers. A reduction in *NOS2* gene expression in CFA-PI was the only statistically significant difference observed (*p < 0.05). Data were compared by Mann–Whitney *U* test between groups within each gene

To investigate whether the dissociation and reaggregation process during CFA-PI formation alters gene expression levels in comparison with intact native islet cells, we conducted qRT-PCR comparing P750 CFA-PI 48 h after formation with native islet control cells. Genes from four categories were assessed: cell communication, secretory function, oxidative stress and apoptosis (ESM Table 1). CFA-PI and native islet controls from eight clinical human donor islet isolations received from three isolation centres were tested. A reduction in *NOS2* gene expression in CFA-PI was the only statistically significant difference observed (Fig. 3).

### CFA-PI are highly functional in vivo

Islet material from six human donors was employed in a marginal-mass transplant model. Samples of native islets, spontaneous aggregates, P1000, P750 and P500 CFA-PI were aliquoted into two doses, 500 IEQ and 1000 IEQ, for transplant under the kidney capsule (ESM Table 8). For all tissue transplanted, DNA content was measured to determine the recovery of native islets post-culture and CFA-PI formation (mean ± SD of 76.3% ± 16.6% vs 74.1% ± 15.9%, respectively) and to confirm that an equivalent mass had been transplanted (ESM Table 9). Although the native islet and CFA-PI groups were not significantly different, the spontaneous aggregate group, as expected, received significantly less material (approximately 0.43 times as much) owing to the low efficiency of spontaneous aggregation. Cell loss during CFA-PI formation was primarily during dissociation (recovery 78.3% ± 11.7%, mean ± SD).

**Fig. 4:**
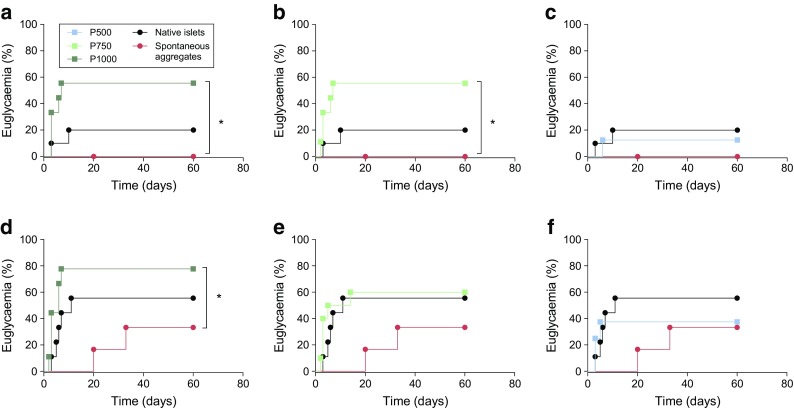
Efficacy of human CFA-PI transplanted into mice at marginal-mass doses (**a**–**c**, 500 IEQ; **d**–**f**, 1000 IEQ). (**a**, **b**) At 500 IEQ, significantly more animals showed a reversal of diabetes (non-fasting blood glucose <11.1 mmol/l) in both the P1000 (*n* = 9) and P750 (*n* = 9) CFA-PI groups compared with the single-cell spontaneous aggregates transplanted (*n* = 6, *p < 0.05). The median reversal time was day 7. They also showed improved diabetes reversal rates (55.6%) over native islets (20.0%, *n* = 10), although this did not reach statistical significance. (**c**) There was no significant difference between the P500 group (*n* = 8) and the native islets (*n* = 10) or spontaneous aggregates (*n* = 6). (**d**) At an increased, marginal dose of 1000 IEQ, the results followed a similar trend, with a significant improvement in diabetes reversal in the P1000 group (*p < 0.05, *n* = 9) compared with the spontaneous aggregates; improvement compared with native islets did not reach statistical significance (*n* = 10). (**e, f**) The proportion of mice showing a reversal of diabetes was similar between the P750 (*n* = 10) and P500 (*n* = 8) CFA-PI groups compared with the native islets (*n* = 9), with lower values for the P500 pseudoislets. Animals were transplanted with *n* = 6 human islet/CFA-PI preparations. Comparison of euglycaemia curves was by Gehan–Breslow–Wilcoxon test. The key above (**a**) applies to all figure parts

At a 500 IEQ marginal-mass transplant dose, significantly more mice showed a reversal of diabetes (defined as maintaining a blood glucose reading <11.1 mmol/l) in both the P1000 and P750 CFA-PI groups when compared with spontaneous aggregates, with a median reversal time of day 7 (Fig. 4a,b). They also appeared to show improved reversal rates over that with native islets, although this effect did not reach statistical significance (55.6% vs 20.0%, *p = 0.09*). There was no significant difference between the P500 group and the groups with native islets and spontaneous aggregates (Fig. 4c). At an increased, marginal dose of 1000 IEQ, the results followed a similar pattern, with a significant improvement in diabetes reversal in the P1000 group compared with spontaneous aggregates, as well as a potential improvement compared with native islets transplanted alone (Fig. 4d), although this failed to reach statistical significance (77.8% vs 55.6%*, p* = 0.13). The proportion of mice showing a reversal of diabetes at 1000 IEQ was similar for the P1000 and P750 CFA-PI and native islets, with the P500 CFA-PI having a potentially somewhat lower reversal rate (Fig. 4d–f).

**Fig. 5:**
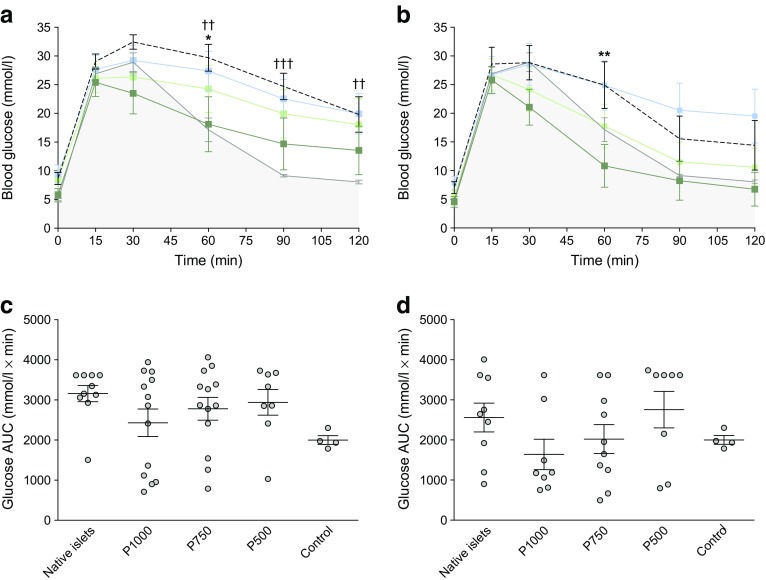
(**a**) At 500 IEQ, glucose clearance was significantly improved in the P1000 group (*n* = 9) compared with the native islet groups (*p < 0.05 at 60 min, *n* = 10). There was significant impairment in the glucose clearance in the mice transplanted with the native islets compared with the naive non-diabetic control mice (††*p* < 0.01 at 60 and 120 min, †††*p* < 0.001 at 90 min, *n* = 4); this impairment was not, however, mirrored in the P1000 group. There was no significant difference between the P750 (*n* = 9) and P500 (*n* = 8) groups at any time during the IPGTT. (**b**) At 1000 IEQ, glucose clearance was significantly improved in the P1000 group at 60 min (**p < 0.01, *n* = 9) compared with the native islet transplant group (*n* = 9). There was no significant difference between the P750 (*n* = 10) and P500 (*n* = 8) groups compared with animals transplanted with native islets at any time during the IPGTT. (**a**, **b**) Dark green line and symbols, P1000; light green line and symbols, P750; blue line and symbols, P500; black dashed line, native islets; grey line, non-diabetic control mice. (**c, d**) When comparing the AUC (**c**, at 500 IEQ; **d**, at 1000 IEQ), differences in overall glucose clearance in the mice transplanted with native islets vs those transplanted with CFA-PI did not reach statistical significance. Mice regardless of euglycaemia were compared (unpaired *t* test between groups at each time point corrected for multiple comparison using the Holm–Sidak method; analysis of AUC by one-way ANOVA with Tukey’s multiple comparisons)

One week before the endpoint of the study, mice were administered an IPGTT. Glucose clearance was significantly improved in the P1000 CFA-PI group compared with the native islet groups transplanted with 500 IEQ (Fig. 5a), although differences reached statistical significance only at the 60 min time point. There was a significant impairment of glucose clearance in the mice transplanted with the native islets compared with the naive non-diabetic control mice; this impairment was not, however, evident in the P1000 CFA-PI group (Fig. 5a). The P750 and P500 CFA-PI groups did not show statistically significant improvement over the native islet control cells at any time point (Fig. 5a). At a higher transplant dose of 1000 IEQ, glucose clearance was significantly improved in the P1000 group at 60 min compared with the native islet group (Fig. 5b). There was no significant difference between the P750 and P500 groups when compared with animals transplanted with native islets at any time during the IPGTT with this higher transplant mass (Fig. 5b). AUC analysis was used to assess overall glucose clearance. Although CFA-PI appeared to show improved overall glucose clearance compared with native islets, these differences did not reach statistical significance because of the high variability associated with human donor material. There was no significant difference in overall glucose clearance in mice transplanted with native islets vs those transplanted with CFA-PI regardless of islet mass transplanted (Fig. 5c,d). As a negative control, animals were also transplanted with spontaneous aggregates (ESM Fig. 8). At 500 IEQ, glucose clearance was significantly improved in the P1000 group compared with the spontaneous aggregate group at several time points. At 1000 IEQ, glucose clearance was similarly significantly improved in both the P1000 and P750 groups compared with the spontaneous aggregate group. There was a significant reduction in AUC in the P1000 group compared with spontaneous aggregation group. As expected, glucose clearance was significantly impaired in the spontaneous aggregate group compared with the non-streptozotocin -treated, non-transplanted euglycaemic control mice at 60, 90 and 120 min at both 500 IEQ and 1000 IEQ.

A subset of harvested grafts was homogenised for analysis of total insulin content. No significant differences in insulin content were observed between CFA-PI and native islet transplanted groups transplanted with the same islet mass (data not shown).

### Analysis of immunohistochemistry of human native islet graft and human pseudoislets grafts post-transplant

**Fig. 6:**
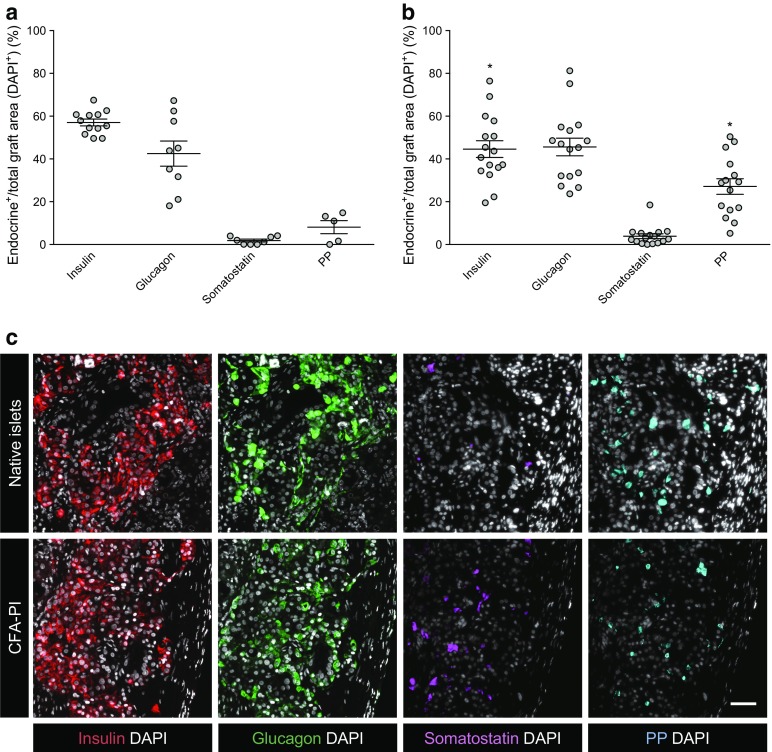
Quantification of the total area of insulin, glucagon, somatostatin and pancreatic polypeptide (PP) immunoreactivity within native islet (**a**) and CFA-PI (**b**) grafts recovered 60 days post-transplant (DAPI^+^ within the graft area, each symbol representing one transplant). Interestingly, despite an equivalent or increased capacity to restore normoglycaemia, CFA-PI grafts showed an increased total PP-positive area and a decreased total insulin-positive area (both *p < 0.05) compared with that of native islet grafts. (**c**) Fluorescent images of human native islet grafts and human CFA-PI grafts stained positive for insulin (red), glucagon (green), somatostatin (magenta), PP (cyan) and nuclei (DAPI, grey) (scale bar, 50 μm). Data from animals transplanted with *n* = 6 independent human islet/CFA-PI preparations. Differences were compared by unpaired *t* test

Immunohistochemistry was performed on a subset of tissue grafts removed 60 days after transplantation. All transplant-bearing kidneys were removed, but no tissue graft could be located on some kidneys transplanted with native islets and spontaneous aggregates at 500 IEQ. Fluorescent staining for major endocrine cell components included insulin, glucagon, somatostatin and pancreatic polypeptide (Fig. 6). There was no obvious difference in overall morphology when comparing the CFA-PI grafts to the native islet groups (Fig. 6c). However, the CFA-PI grafts presented a higher area of pancreatic polypeptide immunoreactivity and a slightly lower area of insulin immunoreactivity (Fig. 6a,b). Two tissue grafts were analysed for the spontaneous aggregate group, and although smaller, the grafts were consistent in composition with both the CFA-PI and native islet grafts (data not shown).

**Fig. 7:**
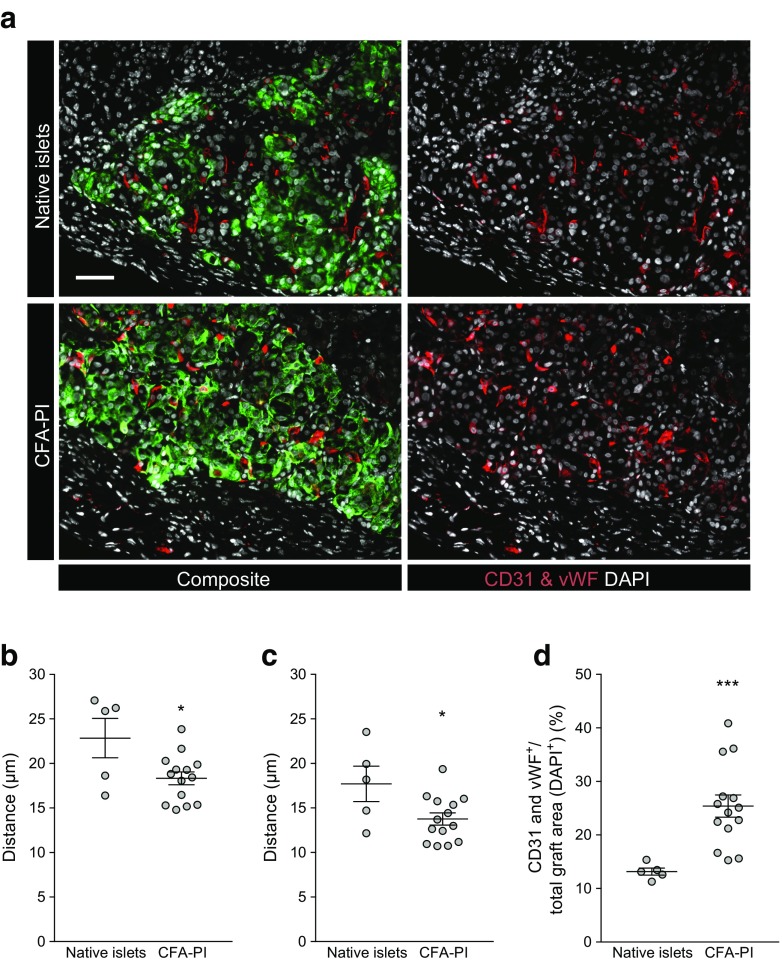
(**a**) Fluorescent images of human native islet and CFA-PI grafts recovered after 60 days and stained for blood vessels (CD31 and von Willebrand factor [vWF], red), insulin (green) and nuclei (DAPI, grey) (scale bar, 50 μm). F-function (distance from 10,000 randomly generated points to the nearest vascular element) results show a 19.8% reduction in mean (**b**) and a 22.3% reduction in median (**c**) distances for CFA-PI grafts, and an increase in overall vascular staining (**d**) compared with native islet grafts. Animals were transplanted with *n* = 6 independent human islet/CFA-PI preparations. *p < 0.05, ***p < 0.001, unpaired *t* test

CD31 and von Willebrand factor combined staining for blood vessels was also performed in the same subset of tissue grafts (Fig. 7a). Quantification of CD31 and von Willebrand factor immunoreactivity within the grafts suggested a significantly higher vessel density of CFA-PI grafts compared with native islet grafts (Fig. 7d). In each image we examined the mean (Fig. 7b) and median (Fig. 7c) distances to the nearest vascular element from randomly selected starting points (*n* = 10,000). CFA-PI grafts showed a significant reduction in both distances, suggesting improved vascular distribution when compared with native islet grafts.

## Discussion

In this study we were able to rapidly, efficiently and reproducibly generate size-controlled CFA-PI from donated human islet material from 21 different donors, obtained from three different distribution programmes across North America. Spontaneous aggregation and hanging-drop methods have been the standard for generating pseudoislets for over a decade [22, 24, 25, 28–30], and more complex strategies (generally employing gravity-mediated settling) have recently been reported. Where efficiency has been reported, it has generally been low due to high cell loss during the process, which would nullify any potential benefit brought about by reaggregation [42, 43]. Our CFA-PI approach enables rapid pseudoislet assembly, minimising the time spent as a single-cell suspension, and efficiently generating uniform, spherical, size-controlled structures whose in vitro survival and functionality was significantly improved over that of the native islets from which they were derived, even after taking into account cell loss during CFA-PI production. Importantly, we are able to complete pseudoislet assembly with minimal loss of islet mass. We attribute this achievement to the rapid sedimentation of islet cells under centrifugation in our process (as opposed to the relatively slow processes inherent in spontaneous aggregation or hanging-drop approaches); they may thus experience only a few minutes as single cells from the moment trituration breaks up the islets to the time they are brought back into contact in the microwells (ESM Fig. 9).

Consistent with modelling studies [17], we observed substantial improvements in both viability and apoptosis in CFA-PI in long-term culture under ambient oxygen levels, which were exaggerated as oxygen levels were further reduced. We also observed a dramatic enhancement of static GSIS secretion over that of unmodified native islets, probably resulting from a combination of enhanced survival, improved function of individual cells due to better oxygen and nutrient access, and improved mass transport of glucose and insulin. The 8.8-fold to 11.1-fold improvement in efficacy ratio (which accounts for cell loss during CFA-PI generation) we observed for CFA-PI therefore represents a significant and substantial improvement in the efficient use of limited donor material. CFA-PI retain function for at least 2 weeks in vitro, confirming that the constituent cells do not suffer from de-differentiation; this is reinforced by the lack of change in gene expression patterns between CFA-PI and native islets. When dealing with three-dimensional structures with a broad size distribution such as islets, it is important to recognise that a consideration of diameter alone tends to exaggerate the contribution of smaller units to the overall population, as volume (and hence proportion of material) increases with the cube of the diameter. While representing only an approximation due to the non-spherical nature of native islets, this can be seen when comparing the estimated mass distributions in Fig. 1f with the diameter distributions from which they are derived. This is consistent with previous reports that have shown better performance from smaller islets [18], while the larger islets, which are inherently less tolerant of hypoxia, generally make up a large proportion of the total islet mass transplanted [44].

In vivo, CFA-PI were examined in terms of ability to rescue hyperglycaemia as well as of short-term glucose clearance, where they performed at least as well as native islets, with hints of potentially even better performance (albeit not statistically significant). In part, this may be simply a reflection of the large variability inherent in both the donor material and the marginal-mass transplant model. We also hypothesise that transplantation under the highly vascularised kidney capsule [45], chosen for the ability to recover the graft, probably provides a conservative model of the improvements that CFA-PI will show in human portal vein delivery. As clinically transplanted islets are believed to undergo a sustained period of hypoxia while trapped within thrombi inside vessels in the liver [46], we expect that the greater hypoxia tolerance exhibited by CFA-PI (Fig. 2d) may provide an added advantage over native islets in this context. Intriguingly, the density of vascular elements (as assessed by dual-staining for CD31 and von Willebrand factor) is significantly higher in CFA-PI (Fig. 7) grafts, with significantly reductions in both the mean and median distances from randomly chosen points to the nearest vascular element (Fig. 7b,c). We hypothesise that this is due to a combination of the relatively smaller size of the CFA-PI, and the fact that their assembly shortly prior to transplantation meant their extracellular matrix was less mature and therefore more easily penetrated by newly forming capillaries. This difference (approximately 20%) is particularly interesting as it represents a shorter path for the transport of essential metabolites (oxygen, nutrients, etc.) required for cell survival, a smaller number of cells competing for those metabolites, and a reduced barrier to both glucose stimulation and insulin secretion. We speculate that this phenomenon may explain the observed changes in glucose clearance (Fig. 5). This phenotype would also be expected to further enhance performance of CFA-PI over native islets in clinical portal vein delivery, where revascularisation of transplanted islets is slow [8] and probably incomplete [11–13]. Much of the performance advantage of CFA-PI is likely to be attributable to a combination of the fact that, in a preparation of native islets, much of the material will be located in larger islets (to a degree that is not always fully appreciated; see Fig. 1f) with the previously reported observations that smaller islets outperform larger ones in clinical applications [17–19].

In conclusion, we have demonstrated the ability to dissociate and reaggregate human islet material efficiently and effectively into engineered CFA-PI, with a high level of consistency, well-controlled size, greatly enhanced hypoxia tolerance and strong in vitro function. The performance of CFA-PI compares favourably with that of native human islets (and is substantially better than conventional spontaneously aggregated pseudoislets) in the mouse kidney capsule, and there are reasons to believe that CFA-PI would perform even better in human portal vein delivery. Although our present efforts have targeted the use of donated human islet material to align with our long-standing experience using this material in the clinic, our CFA-PI process has been designed as a source-agnostic packaging approach, also applicable to material derived from, for example, stem cells [47], cell lines [48, 49] or xenogeneic sources [50], which promises to greatly increase its practical impact. In addition, the ability to generate large numbers of uniform pseudoislets provides a powerful platform to enhance our understanding of islet biology and further optimise performance by assessing the impact of varying cellular compositions and of formation and culture conditions. The ability to genetically modify and manipulate the proportions of different cell types used to form CFA-PI will also provide new opportunities for both basic research and clinical interventions. CFA-PI were formed and transplanted within 72 h, congruent with current clinical islet transplant protocols, and cell loss is not significantly greater than that seen with native islets; in addition, production is not labour intensive and is linearly scalable with microwell surface area. We are in the process of developing microwell bioreactors that will deliver the quantities of CFA-PI required for clinical applications.

## Electronic supplementary material

ESM (PDF 5.38 MB)

## Data Availability

Data are available within the supplementary materials, or on request from the authors.

## Abbreviations

CFA-PI: Pseudoislets engineered via centrifugal-forced-aggregation
GSIS: Glucose-stimulated insulin secretion
IEQ: Islet equivalents
P: Pseudoislet (number; formed from the number of cells indicated)
