# Supplementary material for: Bioengineered human pseudoislets form efficiently from donated tissue, compare favourably with native islets in vitro and restore normoglycaemia in mice
Source: Diabetologia. 2018 Jul 3;61(9):2016–29. doi: 10.1007/s00125-018-4672-5 (PMC6096633; doi:10.1007/s00125-018-4672-5)
Supplement: Supplementary file 1 — (PDF 5.38 MB) [file 125_2018_4672_MOESM1_ESM.pdf]

SUPPLEMENTARY INFORMATION

ESM Methods

Human islet isolation

Human islet preparations were provided by the Alberta Diabetes Institute IsletCore and the Clinical Islet Transplant Program at the University of Alberta in Edmonton with the assistance of the Human Organ Procurement and Exchange (HOPE) program, Trillium Gift of Life Network (TGLN) and Canadian Organ Procurement Organizations (OPO), as well as the NIDDK-funded Integrated Islet Distribution Program (IIDP) at City of Hope, NIH Grant #2UC4DK098085-02, United States. While we saw no evidence of source-specific or purity-specific effects on our findings, *in vivo* experiments were carried out entirely with islets sourced from the ADI IsletCore to maximize consistency and minimize transport effects. *In vitro* experiments were performed in both Calgary (elevation 1,045 m) and Edmonton (elevation 645 m) under approvals for the use of human tissue from the respective Health Research Ethics Boards.

Human islet dissociation and re-aggregation

Islets were suspended in TrypLE Select (Thermo Fisher Scientific, Waltham, MA, USA) and incubated at 37°C in a shaking water bath for 8-10 minutes, followed by trituration to break up remaining clumps. After centrifugation (200g, 2 minutes) the cells were re-suspended in culture medium pre-warmed in a 37ºC water bath. Culture media consisted of CMRL-1066 (Corning, Manassas, VA, USA) supplemented with 1.2 g/l nicotinamide, 10 ml/l ITS, 2 mmol/l $ZnSO_4$, 50 ml/l sodium pyruvate, 10 ml/l Glutamax, 10 ml/l penicillin/streptomycin, 25 ml/l HEPES and 10% FBS (Sigma-Aldrich, Oakville, ON, Canada). CFA-PI were formed in 24-well or 6-well AggreWell 400 plates (STEMCELL Technologies, Vancouver, BC, Canada) as P1000, P750 and P500 (where P1000 represents CFA-PI formed from 1000 cells apiece, P750 from 750 cells, etc.), with centrifugation (200g, 5 minutes) and cultured at density equivalent to 750, 563, 375 IEQ/ml respectively, with one IEQ defined as 1,600 cells [1]. Native islet controls were cultured in 24-well or 6-well ultra-low attachment plates (Corning, Manassas, VA, USA), as were dissociated single cells for spontaneous-aggregation controls, both plated at 500 IEQ/ml and cultured at 37ºC and 5% CO_2 for 3-5 days prior to transplant and further assay. Example comparisons with pseudoislet formation in hanging drops is shown in ESM Figure 9. Medium volumes used were 1ml/well for 24-well plates and 2.5ml/well for 6-well plates.

Static glucose-stimulated insulin secretion (s-GSIS)

An s-GSIS assay was performed for pre-cultured native islets and dissociated single cells, and post 3-5 days of culture for all groups at 200 IEQ/group. The islets were washed of residual glucose three times in glucose free medium, incubated in RPMI-1640 containing low (2.8 mmol/l) glucose for one hour, followed by high (16.7 mmol/l) glucose for an additional hour at 37ºC and 5% CO_2. The supernatant was harvested post glucose incubation and insulin levels measured by ELISA (Cat#10-1113-01, Mercodia, Uppsala, Sweden).

Hypoxic culture and viability analysis

CFA-PI and islets were cultured as described above at 37°C, 5% CO_2 for 7 days in both ambient and hypoxic (5% O_2 incubator) conditions. Islet and CFA-PI viability was then assessed using the inclusion and exclusion dyes fluorescein diacetate (FDA) and propidium iodide (PI) [2]. Islets were aliquoted into a 10 x 35 mm

culture dish containing 460 µl Dulbecco's phosphate buffered saline (DPBS), 10 µl of PI (750 µmol/l in DPBS) and 10 µl of FDA (24 µmol/l in acetone). Imaging by Zeiss AxioObserver Z1 inverted microscope.

Apoptosis TUNEL staining

Apoptosis of islets and pseudoislets was assessed by TUNEL assay (Promega, Madison, WI, USA). Tissue sections were co-stained with anti-insulin antibody at 1:200 concentration (Dako, Mississauga, ON, Canada) and DAPI to identify nuclei. Apoptosis was determined by analyzing the number of positive TUNEL-stained cells as a percentage nuclei within the insulin-positive. Lot #0000267885 passed quality control specifications with HL-60 cells undergoing apoptosis and with fragmented DNA with DNAse where treated cells showed ≥70% cells stained positive in the presence of TdT enzyme and DNASe and ≤10% without. Both a negative and positive section were stained for TUNEL on each slide, to determine specificity. A known positive and negative control slide were used as control. The lot was tested with different tissue types, skin and whole tissue, pancreas and islets embedded in agar.

Real-Time quantitative reverse transcription PCR (qRT-PCR)

RNA was isolated using a Total RNA Purification Kit (Norgen Biotek, Thorold, Canada) and quantified on a NanoDrop 2000 spectrophotometer (Thermo Fisher Scientific) by absorbance at 260 nm. Reverse transcription (RT) was carried out with 1 µg input RNA using the iScript Reverse Transcription Supermix (BioRad, Hercules, USA) per the manufacturer's instructions. Quantitative real-time PCR (qPCR) was carried out on the resulting cDNA diluted 40x with nuclease-free water. Reactions for qPCR were composed of: 4 µl diluted cDNA; 1 µl specific primer pairs (ESM Table 1) at a concentration of 3 µM each; and 5 µl PowerUp SYBR Green Master Mix (Thermo Fisher Scientific). Reactions were carried out on an Applied Biosystems Cycler (Thermo Fisher Scientific) as specified in the manuals provided with the SYBR Green Master Mix. Analysis of qRT-PCR results was performed using the ΔC_t method [3] with normalization against two stable internal reference genes (*POLR2A* and *EIF2B1)*[4].

Islet transplantation

To induce diabetes, female and male adult (8-10 weeks, 20-30 gm) immunodeficient mice (B6.129S7-Rag1$^{tm1Mom/J}$; Jackson Laboratories, Canada) were administered an intraperitoneal injection of streptozotocin (175 mg/kg, Sigma-Aldrich) in acetate phosphate buffer, pH 4.5. Blood glucose levels ≥18 mmol/l for two consecutive readings, were considered diabetic. Exogenous insulin was administered (Linbit® sustained release insulin implant pellet, 0.1U/day, Linshin Canada, Inc.) prior to islet transplantation. Transplantation was within 30 days of diabetes induction. At the time of transplant the insulin pellets were removed and no subsequent insulin therapy was supplied. Animals were bright, alert and active prior to transplant and body weight loss was <10%. Animals that lost >10% of their body weight after transplant and remained hyperglycemic, ≥18 mmol/l, or were not transplanted within 30 days of diabetes induction were electively euthanized. Sample size approximation, n=10 per group, was determined based on previous experiments by the authors using a minimal islet mass transplant model, whereby the predicted difference in reversal of diabetes would be 60% with Type I and Type II Error of Alpha=0.05, Beta=0.2 and Power at 80%.

Native islets, CFA-PI (P500, P750 and P1000), and spontaneous aggregates were transplanted under the capsule of the left kidney [5] at an islet equivalent mass of 500 or 1000 IEQ ± 10% at 90% purity. The islet graft-function was assessed through non-fasting blood glucose measurements, three times per week for 60 days. Blood glucose monitoring was conducted using a portable glucometer (OneTouch Ultra 2, LifeScan, Burnaby, BC, Canada). Two consecutive readings <11.1 mmol/l [5] were considered to confirm

graft function and reversal of diabetes. At day 60, a recovery nephrectomy of the left kidney was performed, and considered to confirm the graft as responsible for diabetes reversal once blood glucose returned to a hyperglycemic state (≥18 mmol/l). Two mice were excluded from the data, one died at day 3 post-transplant, and one did not become hyperglycemic after recovery nephrectomy – data from this mouse was excluded from the analysis due the potential for regeneration of endogenous beta cells [6].

To confirm the islet mass transplanted, islet and CFA-PI DNA content were measured by Quant-iT PicoGreen dsDNA Assay (Invitrogen, Eugene, OR, USA). Islet and CFA-PI aliquots were washed in citrate buffer (150 mmol/l NaCl, 15 mmol/l citrate, 3 mmol/l EDTA, pH 7.4) and stored as cell pellets at -20ºC. Cell pellets were placed in 1 ml of lysis buffer (10 mmol/l Tris, 1 mmol/l EDTA, 0.5% Triton X-100, pH 7.5). Aliquots were assayed in duplicate with 1:5 dilution in TE buffer (10 mmol/l Tris, 1 mmol/l EDTA, pH 7.5). Fluorescence was measured at 485 nm excitation / 525 nm emission.

Intraperitoneal glucose tolerance test (IPGTT)

In vivo glucose tolerance and islet function in mice regardless of euglycemia was assessed by IPGTT 60 days post-transplant. The mice were fasted overnight, and 25% dextrose was administered intraperitoneally at 3 g/kg body weight (Hospira, Lake Forest, IL, USA). Animal group identifications were blinded and blood glucose measurements were monitored at baseline (t=0), 15, 30, 60, 90 and 120 minutes. Mice were tested regardless of euglycemia prior to recovery nephrectomy or euthanasia. Historical controls were utilized in the case where mice were hyperglycemic because of endogenous insulin deficiency and exhibiting signs of mortality.

Immunohistochemistry

Islet and CFA-PI transplant grafts were removed from mice. Immediately after explantation, the kidney bearing the islet graft was fixed in 10% formalin. The tissue was dissected, embedded in paraffin, and sectioned. Following de-paraffinization and antigen heat retrieval, sections were blocked with 20% goat serum (Sigma-Aldrich) in DPBS for 1.5 hours at room temperature. For vessel staining an additional enzymatic antigen retrieval step was performed prior to blocking (Proteinase K at 20 µg/ml for 20 minutes at 37°C). Sections were incubated with primary antibodies overnight at 4°C: guinea pig anti-insulin (Dako, A0564, 1:200), rabbit anti-glucagon (Abcam, ab43837, Cambridge, MA, USA, 1:100), rabbit anti-human pancreatic polypeptide (Abcam, ab14985, 1:100), rat anti-human somatostatin (Abcam, ab30788, 1:100), or rabbit anti-CD31 and anti-von Willebrand factor (vWF) (Abcam, ab124432, 1:50). The following day sections were washed with Tween buffer followed by incubation with secondary antibodies 1:200 for 1 hour at room temperature utilizing goat anti-guinea pig Rhodamine, goat anti-rat fluorescein (Jackson ImmunoResearch, 106-025-003, West Grove, PA, USA), or goat anti-rabbit Alexa Fluro 568 (Abcam). Samples were counterstained with DAPI (Invitrogen, P36931).

Statistical analysis

Data are expressed as mean ± SEM unless otherwise specified. Statistical analyses were performed using GraphPad Prism 7. Imaging was carried out on a Zeiss COLIBRI inverted fluorescence microscope unless otherwise specified and analysis was via ImageJ software (http://rsb.info.nih.gov/ij). Spatial statistical analysis of vascular element distribution was performed using the "Spatial statistics 2D/3D" plugin, implementing the previously published F-function [7, 8], with independent evaluation points set to 10,000, hardcore distance set to 0, pattern samples set to 10.

Antibody validations

Endocrine markers were tested with different tissue types, pancreatic endoderm cells and whole organ, transplanted tissue and islets/pseudoislets. A known positive slide, intact whole islet and pancreas tissue, was used for validation and negative control, kidney tissue, was used in the experiment. Endothelial markers (CD31 and vWF) were tested in both the mouse heart and spleen by western blot and in heart, spleen, kidney and transplant sites with formalin fixed embedded tissue sections. A negative section was included with all slides analyzed, for validation and specificity.

ESM References:

1. Pisania A, Weir GC, O'Neil JJ, et al (2010) Quantitative analysis of cell composition and purity of human pancreatic islet preparations. Lab Invest 90:1661–1675

2. Integrated Islet Distribution Program | IIDP | Human Islets for Research > Investigators > Policies & Standard Operating Procedures. https://iidp.coh.org/Investigators/Policies-Standard-Operating-Procedures. Accessed 5 Dec 2017

3. Livak KJ, Schmittgen TD (2001) Analysis of relative gene expression data using real-time quantitative PCR and the 2(-Delta Delta C(T)) Method. Methods San Diego Calif 25:402–408

4. Vandesompele J, De Preter K, Pattyn F, et al (2002) Accurate normalization of real-time quantitative RT-PCR data by geometric averaging of multiple internal control genes. Genome Biol 3:RESEARCH0034

5. Pepper AR, Gala-Lopez B, Pawlick R, et al (2015) A prevascularized subcutaneous device-less site for islet and cellular transplantation. Nat Biotechnol 33:518

6. King AJ (2012) The use of animal models in diabetes research. Br J Pharmacol 166:877–894

7. Andrey P, Kiêu K, Kress C, et al (2010) Statistical Analysis of 3D Images Detects Regular Spatial Distributions of Centromeres and Chromocenters in Animal and Plant Nuclei. PLOS Comput Biol 6:e1000853

8. Ollion J, Cochennec J, Loll F, et al (2013) TANGO: a generic tool for high-throughput 3D image analysis for studying nuclear organization. Bioinformatics 29:1840–1841

ESM Table 1: Human gene primers for real-time qPCR

Gene symbol	Alias/common name	Primer sequence (5'-3')
Referencing genes		
POLR2A	RNA Polymerase II Subunit A	F-TCACAGCAGTGCGCAAATTC
		R-CCACGTCGACAGGAACATCA
EIF2B1	Eukaryotic Translation Initiation Factor 2B Subunit Alpha	F-CGGACGTTGCTGGAGTTCTT
		R-CCACACCACACAGGGTTTCT
Oxidative defence		
SOD1	Superoxide dismutase 1	F-GGTGGGCCAAAGGATGAAGAG
		R-CCACAAGCCAAACGACTTCC
SOD2	Superoxide dismutase 2	F-TCCCAAGGGAAACACTCGGCTTT
		R-AAACCACTGGGTGACATCTACC
CAT	Catalase	F-CGTGCTGAATGAGGAACAGA
		R-AGTCAGGGTGGACCTCAGTG
Secretory function		
INS	Insulin	F-GGCCTTTGCGTCAGATCACTG
		R-GTTCCCCGCACACTAGGTAGA
PDX1	Pancreatic and duodenal homeobox 1, insulin promoter factor 1	F-GGGAAAACCCGCTCTCTCAG
		R-CCAAGGTGGAGTGCTGTAGG
GLP1R	Glucagon Like Peptide 1 Receptor	F-TTGTGAAACCACAGGCCCTT
		R-CTTGCAAGCCCCAGTTTCAC
PCSK1	Proprotein Convertase Subtilisin/Kexin Type 1	F-GCCGAACTGACTATGGGGAA
		R-AAAGGCACTCCTTCGAGACC
PCSK2	Proprotein Convertase Subtilisin/Kexin Type 2	F-GTGTGTTTGCACTGGCTCTG
		R-TTAAATTCCAGGCCGACCCC
Cell communication		
GJA1	Connexin 43, gap junction protein alpha 1	F-CAATCTCTCATGTGCGCTTCT
		R-GGCAACCTTGAGTTCTTCCTCT
CDH1	E-Cadherin	F-GCTGGACCGAGAGAGTTTCC
		R-CGACGTTAGCCTCGTTCTCA
LAMB1	Laminin Subunit Beta 1	F-AAAAGACATCCTGGCGCAGA
		R-TTCTTTGGCTGTGCTGTTGC
ITGB1	Integrin Subunit Beta 1	F-GCCGCGCGGAAAAGATGAAT

		R-ACATCGTGCAGAAGTAGGCA
ITGB7	Integrin Subunit Beta 7	F-AGAATGGCGGAATCCTCACCT
		R-TGAAGTTCAGTTGCTTGCACC
Apoptosis		
NFKB1	Nuclear Factor of Kappa Light Polypeptide Gene Enhancer In B-Cells 1	F-AACAGAGAGGATTTCGTTTCCG
		R-TTTGACCTGAGGGTAAGACTTCT
NOS2	Inducible Nitric Oxide Synthase	F-CCCACCAGACAGTGCGCCTG
		R-GGAGCAGCAGCTGGGTTGGG
NOS3	Endothelial Nitric Oxide Synthase	F-GTGGCTGGTACATGAGCACT
		R-GTGGTCCACGATGGTGACTT
MAPK8	Mitogen-Activated Protein Kinase 8	F-TGTGTGGAATCAAGCACCTTC
		R-AGGCGTCATCATAAAACTCGTTC
MAPK10	Mitogen-Activated Protein Kinase 10	F-CTGGTATGACCCAGCCGAAG
		R-GCACCTGTGCTGAAGGAGAA
APAF1	Apoptotic Peptidase Activating Factor 1	F-TCCAGTCCAGGTTTCAGCAC
		R-CTGTTTCCTGATGGCCTCGT

ESM Table 2: Summary of in vitro parameters and in vivo outcomes of native islets and corresponding CFA-PIs by donor islet preparations used both in vitro and in vivo.

Stimulation Index (SI)

	Native islets		Spontaneous aggregates		P1000		P750		P500	
	mean	SEM	mean	SEM	mean	SEM	mean	SEM	mean	SEM
**R200**	2.06	0.51	1.22	0.44	1.81	0.56	2.65	0.14	1.86	0.95
**R201**	0.78	0.19	0.70	0.06	1.17	0.20	1.80	0.57	0.77	0.10
**R202**	1.43	0.11	1.22	0.28	1.74	0.57	1.62	0.28	1.53	0.37
**R226**	0.37	0.65	1.16	0.30	9.04	1.54	11.07	2.07	6.37	1.41
**R227**	1.89	0.90	1.76	0.59	9.18	1.21	7.30	0.95	5.99	1.08

Efficacy Ratio (fg / input cell)

	Native islets		Spontaneous aggregates		P1000		P750		P500	
	mean	SEM	mean	SEM	mean	SEM	mean	SEM	mean	SEM
**R200**	66.50	4.48	10.79	31.35	76.10	42.53	121.20	16.74	61.85	74.46
**R201**	-59.14	42.87	-66.18	13.40	16.60	34.35	86.62	67.20	-57.11	32.43
**R202**	27.02	4.01	6.22	11.48	17.98	12.95	20.51	6.87	12.80	7.84
**R226**	-177.40	77.37	6.06	40.72	514.10	102.30	652.60	131.10	477.40	79.22
**R227**	67.74	68.79	101.00	128.50	1204.00	93.11	1074.00	238.70	449.90	55.17

GSIS (fg / incorporated cell)

	Native islets		Spontaneous aggregates		P1000		P750		P500	
	mean	SEM	mean	SEM	mean	SEM	mean	SEM	mean	SEM
**R200**	89.90	6.06	26.93	78.29	91.15	50.94	170.70	23.58	93.20	112.20
**R201**	-67.97	49.27	-263.90	53.42	16.24	33.60	82.22	63.78	-55.53	31.54
**R202**	30.46	4.52	32.17	59.39	20.55	14.81	25.02	8.38	14.62	8.95
**R226**	-191.60	83.58	8.78	59.02	505.00	100.50	607.60	122.10	427.20	70.90
**R227**	126.10	128.10	222.30	282.70	1149.00	88.86	991.30	220.40	552.00	67.70

Basal secretion (ER) (fg / input cell)

	Native islets		Spontaneous aggregates		P1000		P750		P500	
	mean	SEM	mean	SEM	mean	SEM	mean	SEM	mean	SEM
**R200**	106.57	50.46	81.63	7.75	114.60	17.52	74.86	16.50	105.31	29.56
**R201**	188.90	50.80	217.74	4.03	158.53	31.60	144.45	24.73	227.76	33.52
**R202**	70.91	16.96	41.90	6.05	30.21	5.45	36.69	4.00	30.24	4.69
**R226**	347.41	67.65	152.93	36.16	64.50	6.95	64.88	5.00	98.36	21.21
**R227**	83.36	3.77	275.50	93.02	152.71	20.49	167.81	14.30	96.03	15.45

Stimulated secretion (ER) (fg / input cell)

	Native islets		Spontaneous aggregates		P1000		P750		P500	
	mean	SEM	mean	SEM	mean	SEM	mean	SEM	mean	SEM
**R200**	173.07	54.01	92.41	23.60	190.70	30.59	196.03	33.24	167.16	44.89
**R201**	129.77	9.25	151.56	13.28	175.13	24.22	231.07	42.86	170.65	11.47
**R202**	97.93	18.06	48.12	7.18	48.19	10.06	57.20	2.97	43.05	4.48
**R226**	170.00	29.99	158.99	15.31	578.63	104.28	717.50	132.71	575.75	88.09
**R227**	151.10	65.25	376.54	35.79	1357.06	99.53	1241.48	250.93	545.88	48.17

Basal secretion (GSIS) (fg / incorporated cell)

	Native islets		Spontaneous aggregates		P1000		P750		P500	
	mean	SEM	mean	SEM	mean	SEM	mean	SEM	mean	SEM
**R200**	144.07	68.22	203.84	19.36	137.26	20.98	105.49	23.25	158.67	44.55
**R201**	217.11	58.38	868.13	16.05	155.08	30.92	137.10	23.47	221.49	32.60
**R202**	79.94	19.12	216.74	31.30	34.53	6.23	44.74	4.87	34.54	5.35
**R226**	375.28	73.08	221.66	52.41	63.36	6.83	60.41	4.65	88.02	18.98
**R227**	155.21	7.02	606.09	204.63	145.75	19.56	154.94	13.20	117.84	18.96

Stimulated secretion (GSIS) (fg / incorporated cell)

	Native islets		Spontaneous aggregates		P1000		P750		P500	
	mean	SEM	mean	SEM	mean	SEM	mean	SEM	mean	SEM
**R200**	233.96	73.01	230.78	58.94	228.40	36.64	276.22	46.83	251.87	67.64
**R201**	149.15	10.63	604.27	52.93	171.32	23.70	219.32	40.68	165.96	11.15
**R202**	110.39	20.36	248.91	37.14	55.08	11.50	69.76	3.62	49.16	5.12
**R226**	183.64	32.39	230.44	22.19	568.38	102.44	668.06	123.56	515.24	78.83
**R227**	281.34	121.49	828.38	78.75	1295.19	94.99	1146.29	231.69	669.88	59.11

In vivo reversal rate % (reversed / transplanted) - 500 IEQ

	Native islets	Spontaneous aggregates	P1000	P750	P500
**R200**	0% (0/1)	0% (0/1)	0% (0/1)	0% (0/1)	0% (0/1)
**R201**	0% (0/2)	0% (0/2)	0% (0/2)	0% (0/3)	0% (0/2)
**R202**	100% (1/1)	0% (0/1)	66.6% (2/3)	100% (2/2)	50% (1/2)
**R226**	0% (0/3)	0% (0/1)	100% (2/2)	100% (1/1)	0% (0/3)
**R227**	0% (0/2)	0% (0/1)	-	100% (1/1)	100% (1/1)

In vivo reversal rate % (reversed / transplanted) - 1000 IEQ

	Native islets	Spontaneous aggregates	P1000	P750	P500
**R200**	-	-	-	-	-
**R201**	0% (0/1)	0% (0/1)	0% (0/1)	0% (0/1)	-
**R202**	100% (1/1)	100% (1/1)	100% 2/2)	50% (1/2)	50% (1/2)
**R226**	100% (3/3)	100% (1/1)	100% (3/3)	100% (2/2)	50% (2/4)
**R227**	33.3% (1/3)	0% (0/3)	66.6% (2/3)	50% (2/4)	0% (0/0)

ESM Table 3: In vitro parameters of individual samples tested in donor preparation R200

R200	*Stimulation Index*	*Basal (fg/input cell)*	*Stimulated (fg/input cell)*	*Efficacy Ratio (fg/input cell)*	*GSIS (fg/incorporated cell)*	*GSIS basal (fg /incorporated cell)*	*GSIS stimulated (fg /incorporated cell)*
Native Islets	1.78	90.83	161.83	71.00	95.99	122.79	218.78
	3.05	28.10	85.64	57.54	77.78	37.99	115.78
	1.35	200.77	271.72	70.95	95.92	271.42	367.33
Spontaneous aggregates	0.69	90.83	62.83	-27.99	-69.91	226.82	156.91
	2.10	66.22	139.06	72.84	181.91	165.37	347.28
	0.86	87.83	75.34	-12.49	-31.20	219.34	188.14
P1000	2.92	82.04	239.92	157.88	189.09	98.26	287.35
	1.13	119.67	134.63	14.96	17.92	143.33	161.25
	1.39	142.09	197.56	55.47	66.43	170.18	236.61
P750	2.79	58.36	162.79	104.43	147.15	82.24	229.39
	2.51	91.36	229.27	137.90	194.32	128.74	323.05
P500	0.91	134.87	122.27	-12.60	-18.99	203.22	184.23
	2.80	75.74	212.05	136.31	205.38	114.12	319.51

ESM Table 4: In vitro parameters of individual samples tested in donor preparation R201

R201	*Stimulation Index*	*Basal (fg/input cell)*	*Stimulated (fg/input cell)*	*Efficacy Ratio (fg/input cell)*	*GSIS (fg/incorporated cell)*	*GSIS basal (fg /incorporated cell)*	*GSIS stimulated (fg /incorporated cell)*
Native Islets	0.53	279.98	148.26	-131.72	-151.39	321.79	170.40
	1.16	104.39	121.07	16.68	19.17	119.98	139.15
	0.66	182.33	119.97	-62.36	-71.67	209.56	137.89
Spontaneous aggregates	0.73	225.33	163.39	-61.94	-246.96	898.39	651.42
	0.79	211.62	166.24	-45.38	-180.93	843.72	662.78
	0.58	216.28	125.06	-91.22	-363.67	862.28	498.61
P1000	1.49	146.83	218.66	71.83	70.27	143.64	213.90
	1.22	110.59	134.94	24.36	23.83	108.18	132.01
	0.79	218.17	171.78	-46.39	-45.38	213.43	168.05
P750	2.76	104.27	287.83	183.55	174.22	98.97	273.19
	0.78	189.53	147.07	-42.46	-40.30	179.89	139.59
	1.85	139.55	258.32	118.78	112.73	132.45	245.18
P500	0.91	209.85	190.35	-19.50	-18.97	204.08	185.11
	0.58	292.66	170.98	-121.68	-118.33	284.60	166.28
	0.83	180.77	150.63	-30.14	-29.31	175.79	146.48

ESM Table 5: In vitro parameters of individual samples tested in donor preparation R202

R202	*Stimulation Index*	*Basal (fg/input cell)*	*Stimulated (fg/input cell)*	*Efficacy Ratio (fg/input cell)*	*GSIS (fg/incorporated cell)*	*GSIS basal (fg /incorporated cell)*	*GSIS stimulated (fg /incorporated cell)*
Native Islets	1.44	79.00	114.01	35.01	39.47	89.06	128.53
	1.61	38.34	61.88	23.54	26.54	43.22	69.76
	1.24	95.38	117.88	22.50	25.36	107.53	132.89
Spontaneous aggregates	0.75	53.99	40.30	-13.69	-70.82	279.27	208.45
	1.72	36.38	62.46	26.08	134.90	188.18	323.08
	1.18	35.33	41.60	6.27	32.42	182.77	215.19
P1000	1.25	26.36	32.84	6.47	7.40	30.13	37.54
	2.88	23.30	67.14	43.83	50.11	26.64	76.75
	1.09	40.96	44.59	3.63	4.14	46.82	50.97
P750	1.34	41.45	55.39	13.94	17.00	50.56	67.55
	1.34	39.86	53.21	13.35	16.29	48.61	64.89
	2.19	28.75	63.00	34.25	41.77	35.06	76.83
P500	0.96	35.56	34.15	-1.41	-1.61	40.62	39.01
	2.23	20.90	46.53	25.62	29.27	23.87	53.14
	1.41	34.26	48.46	14.19	16.21	39.14	55.35

ESM Table 6: In vitro parameters of individual samples tested in donor preparation R226

R226	*Stimulation Index*	*Basal (fg/input cell)*	*Stimulated (fg/input cell)*	*Efficacy Ratio (fg/input cell)*	*GSIS (fg/incorporated cell)*	*GSIS basal (fg /incorporated cell)*	*GSIS stimulated (fg /incorporated cell)*
Native Islets	0.65	350.64	229.30	-121.34	-131.07	378.77	247.70
	0.29	462.94	132.59	-330.35	-356.85	500.07	143.23
	0.65	228.66	148.11	-80.54	-87.01	247.00	159.99
Spontaneous aggregates	0.72	224.33	160.70	-63.63	-92.22	325.14	232.92
	1.72	107.22	184.62	77.39	112.17	155.41	267.58
	1.03	127.25	131.66	4.41	6.39	184.43	190.82
P1000	7.04	76.67	539.81	463.14	454.93	75.31	530.24
	12.07	64.24	775.51	711.27	698.66	63.11	761.77
	8.00	52.59	420.57	367.98	361.46	51.66	413.12
P750	10.30	74.77	770.24	695.46	647.54	69.62	717.16
	14.98	61.16	916.39	855.23	796.30	56.95	853.25
	7.94	58.71	465.86	407.15	379.09	54.66	433.76
P500	4.11	114.96	472.65	357.69	320.09	102.88	422.97
	6.06	123.87	751.01	627.15	561.23	110.85	672.08
	8.95	56.25	503.59	447.34	400.32	50.34	450.66

ESM Table 7: In vitro parameters of individual samples tested in donor preparation R227

R227	*Stimulation Index*	*Basal (fg/input cell)*	*Stimulated (fg/input cell)*	*Efficacy Ratio (fg/input cell)*	*GSIS (fg/incorporated cell)*	*GSIS basal (fg /incorporated cell)*	*GSIS stimulated (fg /incorporated cell)*
Native Islets	0.96	89.2	85.6	-3.61	-6.72	166.09	159.37
	1.02	84.56	86.11	1.55	2.88	157.45	160.33
	3.69	76.31	281.6	205.29	382.23	142.09	524.32
Spontaneous aggregates	0.67	457.94	305.01	-152.93	-336.45	1007.45	671
	1.9	215.78	409.89	194.11	427.04	474.71	901.74
	2.71	152.77	414.72	261.95	576.29	336.1	912.38
P1000	7.12	175.72	1251.9	1076.18	1027.12	167.71	1194.83
	9.12	170.58	1556.01	1385.43	1322.27	162.8	1485.07
	11.3	111.83	1263.26	1151.43	1098.94	106.73	1205.67
P750	5.62	162.47	912.58	750.11	692.6	150.01	842.61
	7.37	146.16	1077.63	931.48	860.06	134.95	995.01
	8.9	194.8	1734.21	1539.41	1421.38	179.87	1601.25
P500	6.36	77.34	492.29	414.95	509.2	94.91	604.12
	3.97	126.69	503.35	376.66	462.22	155.47	617.69
	7.64	84.05	642	557.95	684.69	103.14	787.83

ESM Table 8: Diabetes reversal days and transplant distribution.

	500 IEQ					1000 IEQ				
Cohort	Native islets (n=10)	Spontaneous aggregates (n=6)	P500 (n=8)	P750 (n=9)	P1000 (n=9)	Native islets (n=9)	Spontaneous aggregates (n=6)	P500 (n=8)	P750 (n=10)	P1000 (n=9)
R190	3			3		3			3	
R200	X	X	X	X	X					
R201	X, X	X, X	X, X	X, X, X	X, X	X	X		X	X
R202	10	X	6, X	3, 6	3, 6, X	6	20	3, X	3, X	6, 6
R226	X, X, X	X	X, X, X	7	3, 3	5, 7, 11	33	3, 5, X, X	3, 5	3, 3, 3
R227	X, X	X		2	7	X, X, 7	X, X, X	X, X	2, 14, X, X	2, 7, X

Human islet donor cohorts are assigned a unique R= research identification number prior to tissue release. IEQ = islet equivalents. Numbers shown in the table represent the number of days it took for each animal to reach euglycemia. X represents animals that did not reach euglycemia.

ESM Table 9: Assessment of genomic DNA per IEQ.

	Native islets		CFA-PI		Spontaneous aggregates	
	mean	SD	mean	SD	mean	SD
Prior to dissociation (µg)	10.77	1.23	12.08	1.74	12.08	1.74
Post dissociation (µg)	-	-	9.45	0.39	9.45	0.39
Post culture/pre transplantation (µg)	8.21	1.52	8.95	1.43	3.82	1.87
Dissociation recovery rate	-	-	78.3%	11.7%	78.3%	11.7%
Overall recovery rate	76.3%	16.6%	74.1%	15.9%	31.6%	16.1%

SUPPLEMENTARY FIGURES

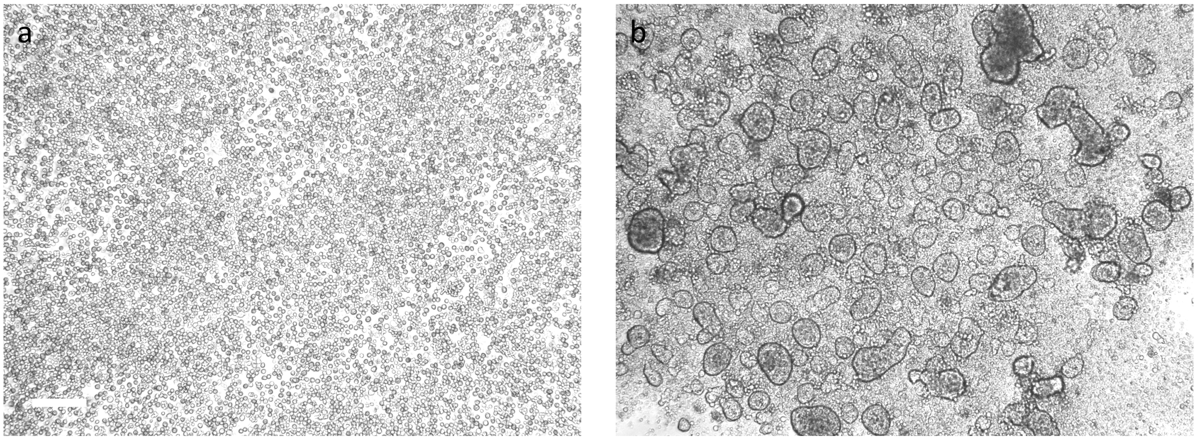

ESM Fig. 1 Pancreatic cell suspensions immediately post-dissociation (a) appear uniformly as single cells. The single-cell suspensions were cultured on ultra-low attachment plates for conventional spontaneous aggregate formation, visible here after 96 hours in culture - note heterogeneous size distribution with a substantial proportion of single cells and debris (b). Scale bar represents 200 µm.

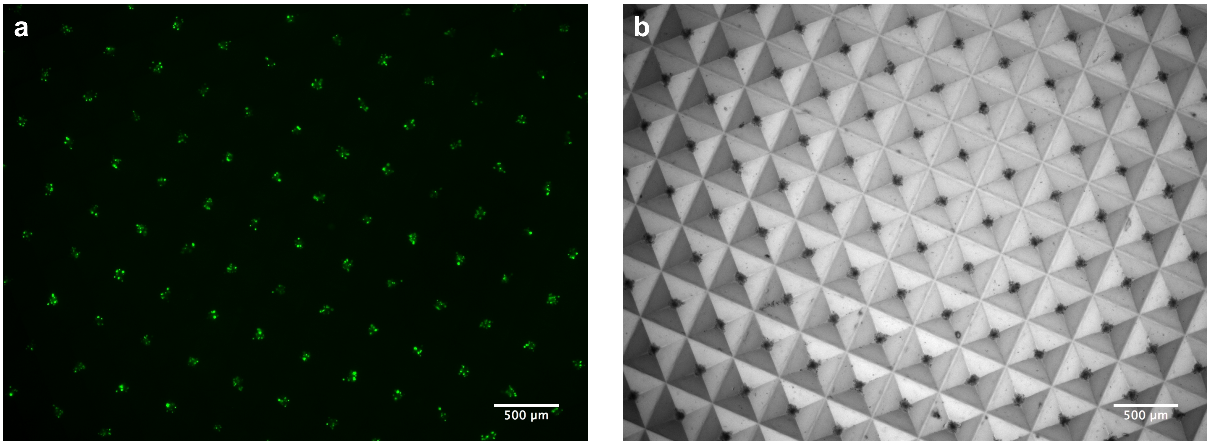

ESM Fig. 2 CFA-PI in Aggrewell 48 hours after GFP transfection at dissociated single cells stage prior to formation, showing GFP fluorescence retained in CFA-PI (a) and its bright phase (b). Scale bars represent 500 µm.

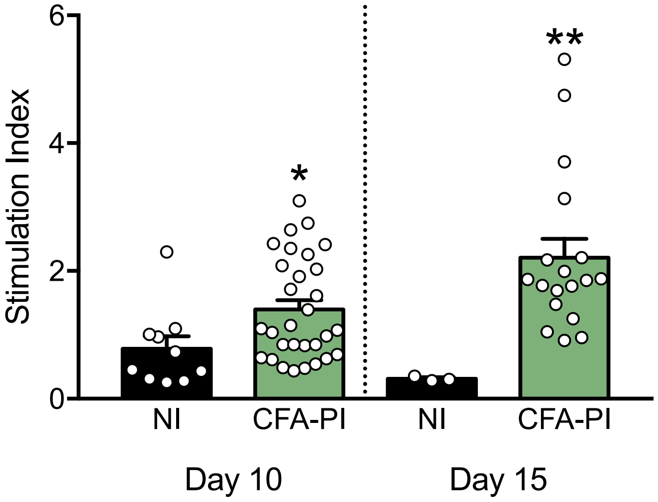

ESM Fig. 3 The Stimulation Index shows increased tolerance for long term culture by CFA-PI (P1000, P750 and P500) as compared to native islets. At both day 10 and day 15 post-culture, CFA-PI retained a significantly higher simulation index compared to the native islets from which they were formed ($n \geq 10$ at day 10, $n \geq 3$ at day 15; $^{**}p<0.01$, $^{*}p<0.05$, Mann-Whitney *U* test).

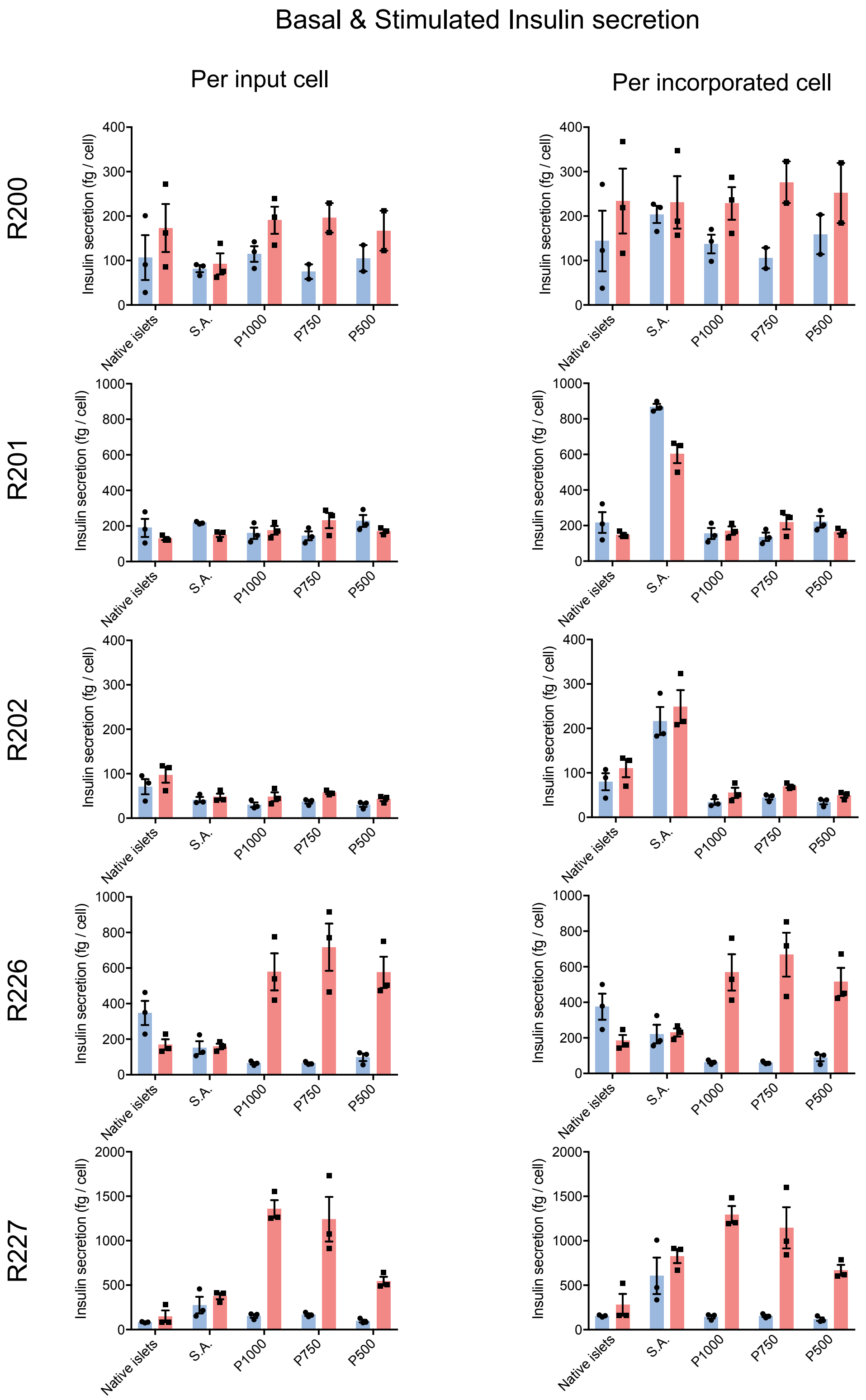

ESM Fig. 4 Basal (at 2.8 mmol/l glucose, blue) and stimulated (at 16.7 mmol/l glucose, red) insulin secretion of native islets, spontaneous aggregates (S.A.) and CFA-PIs broken out by donor, normalized to the amount of material (left) present prior to dissociation and re-aggregation (Efficacy ratio); and (right) present at time of assay (traditional normalization for Glucose Stimulated Insulin Secretion).

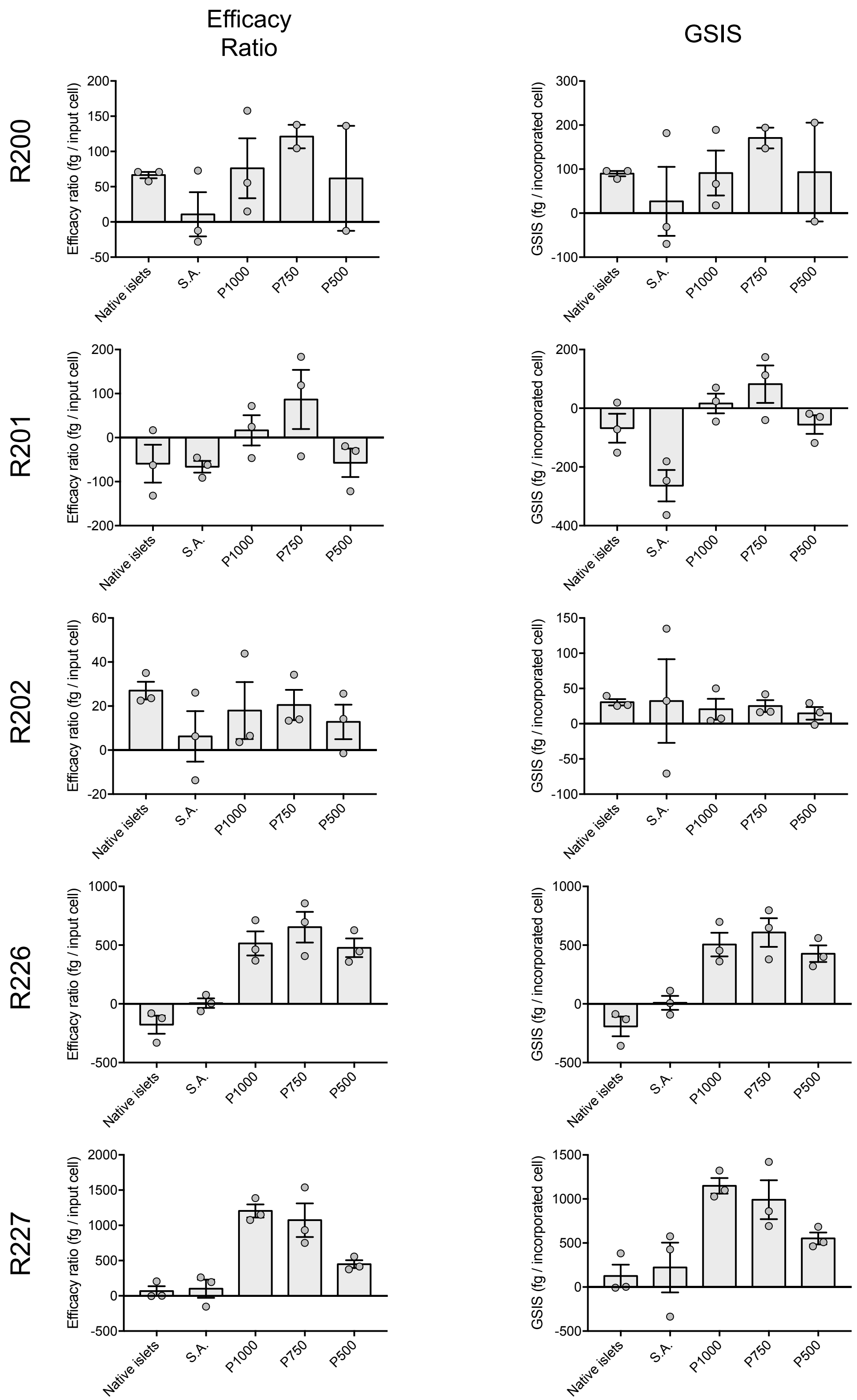

ESM Fig. 5 Efficacy ratio and GSIS of native islets, spontaneous aggregates (S.A.) and CFA-PIs broken out by donor, calculated from the basal and stimulated insulin secretion data shown in ESM Fig. 4.

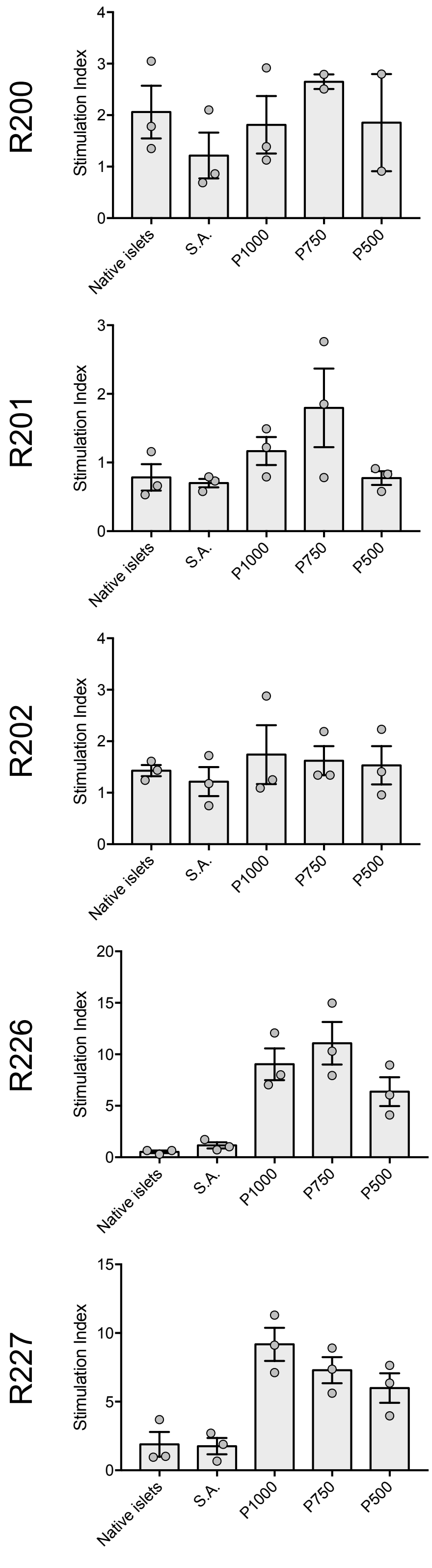

ESM Fig. 6 Stimulation Index of native islets, spontaneous aggregates (S.A.) and CFA-PIs broken out by donor, calculated from the basal and stimulated insulin secretion data shown in ESM Fig. 4.

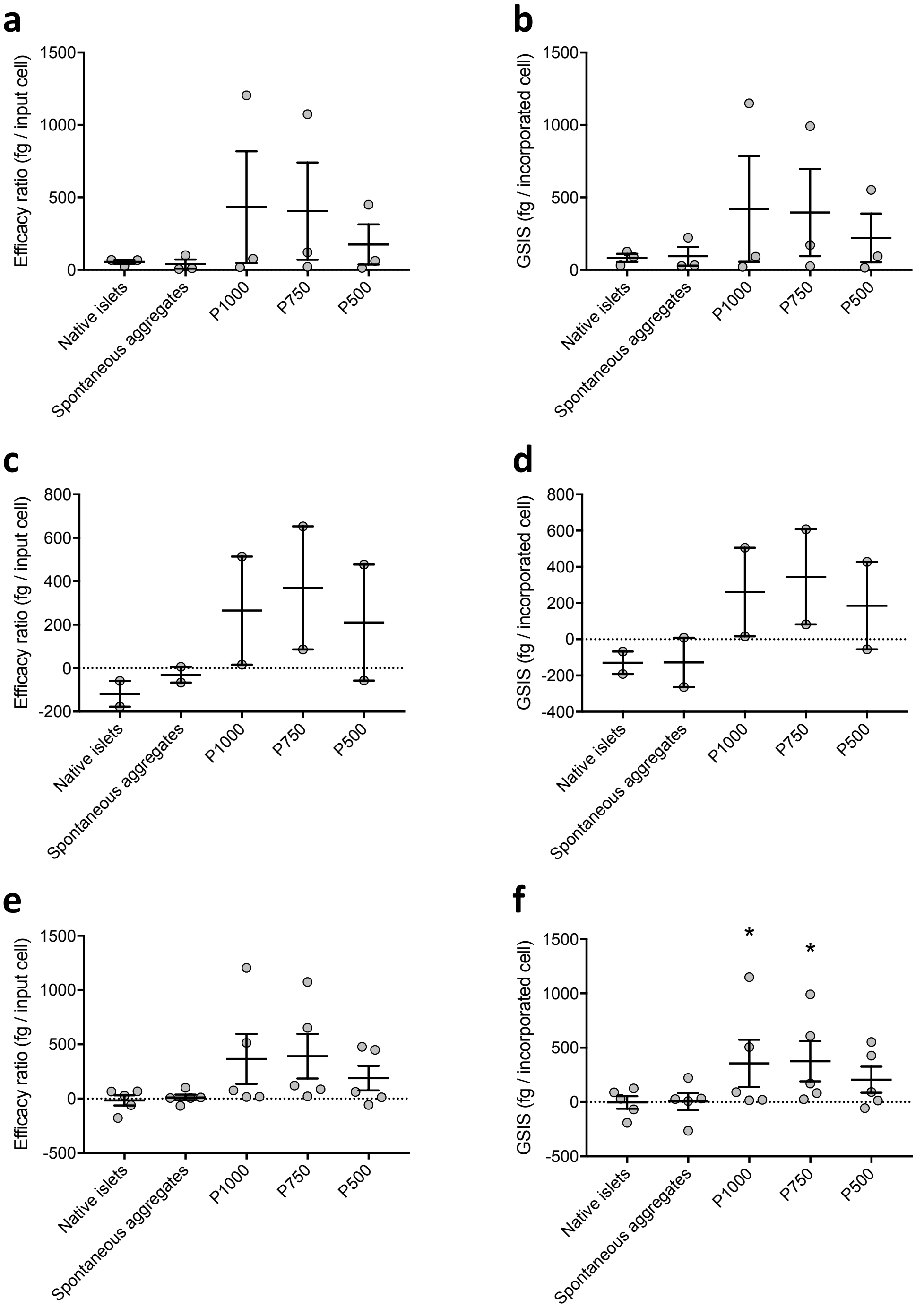

ESM Fig. 7 Overview of Efficacy ratio and GSIS results for native islets, spontaneous aggregates and CFA-PIs either divided into subgroups of "high-function" islet preparations (native islet controls exhibit Stimulation Index > 1) (a, b respectively) and "low-function" islet preparations (native islet controls exhibit Stimulation Index < 1) (c, d respectively); or pooled (e, f respectively). Paired and matched one-way ANOVA with Holm-Sidak's multiple comparison analysis has been performed for both e and f. GSIS for P1000 and P750 is significantly improved over native islets, with *$p<0.05$ (f). Corresponding Efficacy ratio values for P1000 and P750 also appear increased, although this difference does not reach statistical significance (p=0.0746, p=0.0714, respectively) (e).

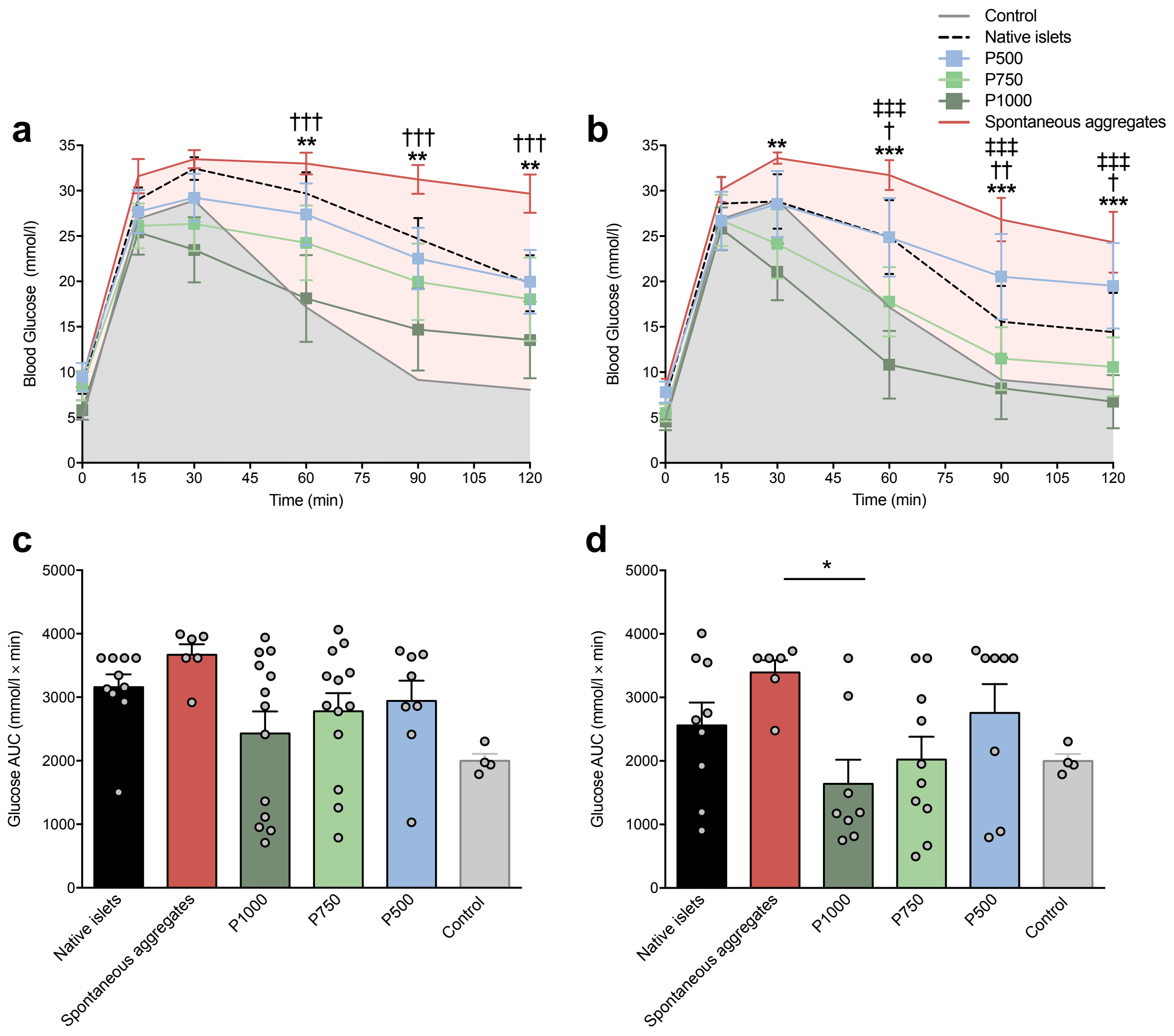

ESM Fig. 8 As a negative control, animals were also transplanted with spontaneous aggregates. At 500 IEQ, glucose clearance was significantly improved in the P1000 group, n=8, compared to the spontaneous aggregates, n=6 (***p*<0.01 at 60, 90 and 120 minutes) (a). There was no significant difference in the P750 group, n=9 and P500 group, n=8. At 1000 IEQ, glucose clearance was markedly improved within both the P1000, n=9, and P750 groups, n=10 (***p*<0.01 at 30 minutes and ****p*<0.001 at 60, 90 and 120 minutes; †*p*<0.05 at 60 and 120 minutes and ††*p*<0.01 at 90 minutes, respectively) (b). There were no significant differences between groups transplanted with 500 IEQ when comparing areas under the curve (c). There was significantly improved overall clearance in the P1000 group transplanted with 1000 IEQ when comparing the areas under the curve (**p*<0.05) (d). As expected, naïve non-transplanted euglycemic control were significantly different from the spontaneous aggregate transplanted mice at 60, 90 and 120 minutes for both 500 IEQ and 1000 IEQ islet mass' (†††*p*<0.001, ‡‡‡ *p*<0.001) (unpaired *t* test between groups at each time point corrected for multiple comparison using the Holm-Sidak method; analysis of AUC by one-way ANOVA with Tukey's multiple comparisons).

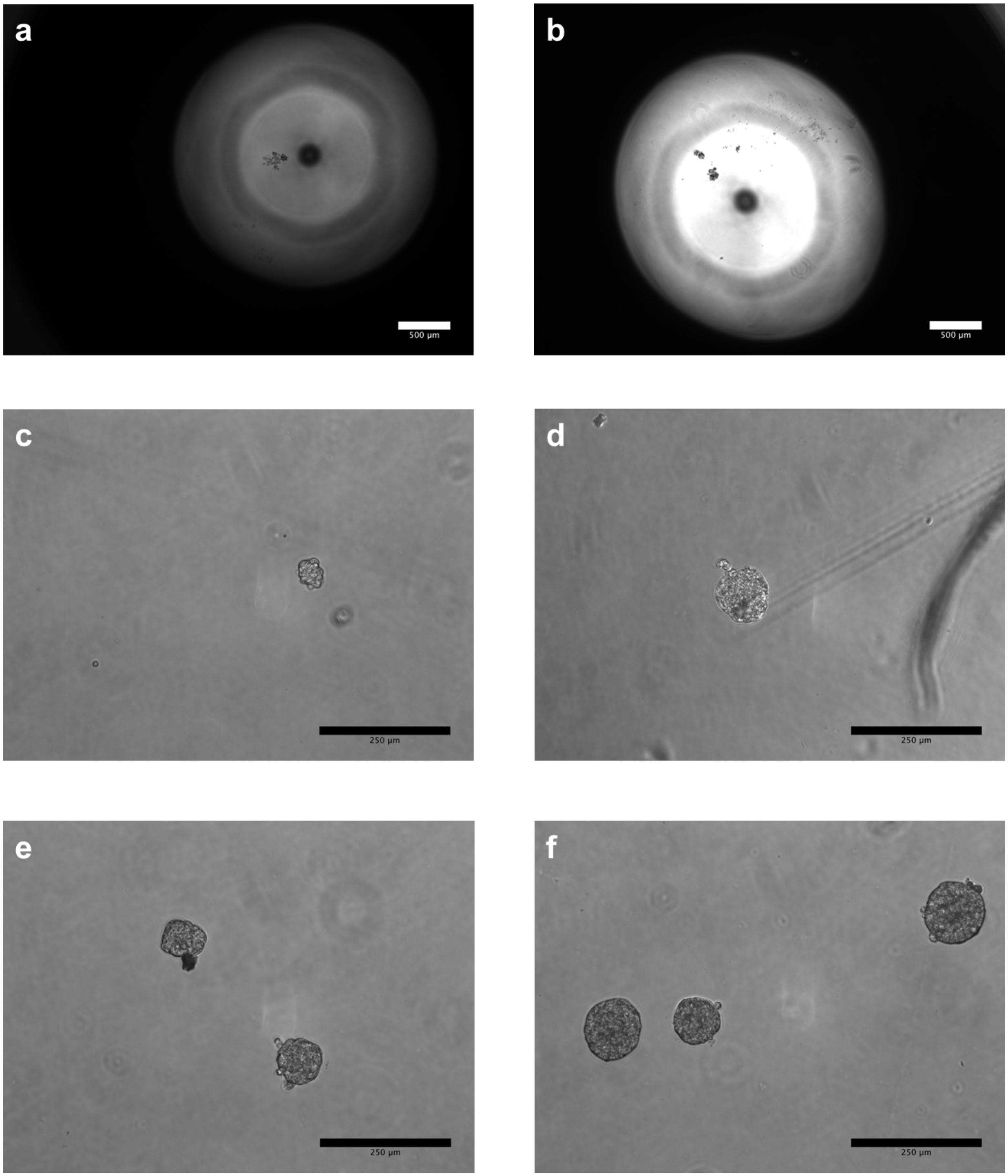

ESM Fig. 9 Comparison of pseudoislets formed via hanging drop and CFA-PI approaches, 5 days post initiation. Pseudoislets were initiated from 250 (a, c, e) or 750 (b, d, f) cells apiece, and representative images are shown inside (a, b), or after extraction from the hanging drop (c, d), alongside CFA-PI controls formed from the same donor islet preparation (e, f). CFA-PI were larger and more cohesive, likely as a result of accelerated clustering (and therefore reduced time as isolated single cells). Scale bars represent 250 µm.
